# Supplementary material for: Tools for mapping multi-scale settlement patterns of building footprints: An introduction to the R package foot
Source: PLoS One. 2021 Feb 25;16(2):e0247535. doi: 10.1371/journal.pone.0247535 (PMC7906393; doi:10.1371/journal.pone.0247535)
Supplement: S1 Text — (DOCX) [file pone.0247535.s001.docx]

**Supplemental Materials for “Tools for mapping multi-scale settlement patterns of building footprints: An introduction to the R package *foot*”**

**Installation instructions**

The *foot* package is available through Github on the website: <https://github.com/wpgp/foot> . The package, documentation, and other dependencies can be installed using the *devtools* package within R:

devtools::install_github("wgpg/foot", build_vignettes=TRUE)
