# Supplementary material for: Tools for mapping multi-scale settlement patterns of building footprints: An introduction to the R package foot
Source: PLoS One. 2021 Feb 25;16(2):e0247535. doi: 10.1371/journal.pone.0247535 (PMC7906393; doi:10.1371/journal.pone.0247535)
Supplement: S2 Text — (PDF) [file pone.0247535.s002.pdf]

Vignette documentation in `foot`

December 10, 2020

Vignettes for the R package `foot`

The `foot` package was developed to support geometric calculations and summaries of morphological measures from building footprint polygons. Copies of the vignettes and documentation from the project are provided here:

1. footsteps: Basic building footprint calculations
2. bigfoot: Gridded building footprint calculation layers
3. cobbler: Using custom summary functions in `foot`

For further documentation, see also: https://wpgp.github.io/foot/

Basic building footprint calculations

December 10, 2020

The `foot` package was developed by WorldPop at the University of Southampton (www.worldpop.org) to provide a set of consistent and flexible tools for processing 2D vector representations of buildings (e.g. "footprints") and calculating urban morphology measurements. The functionality includes basic geometry and morphology characteristics, distance and clustering metrics. These calculations are supported with helper functions for spatial intersections and tiled reading/writing of data.

```
library(foot)
```

This vignette will demonstrate some of the core functionality in the package, including:

- The available measurements and summary statistics
- How to define different types of spatial zones for area-level summaries
- Calculating multiple summary metrics for a set of spatial areas
- Example workflows to produce outputs using `foot::calculate_footstats()`.

To demonstrate the package, this vignette will use a supplied sample of building footprint polygons produced by Microsoft Bing Maps (Source) from an area in Kampala, Uganda. These footprints have been reprocessed into a spatial data format which can be read with `sf`.

Load the sample dataset

```
data("kampala", package = "foot")

buildings <- kampala$buildings
adminzones <- kampala$adminZones
clusters <- kampala$clusters
```

The sample dataset is a list of four named objects:

- "buildings" - polygons of building footprints in `sf` format. Contains 8480 records.
- "mastergrid" - geoTiff `RasterLayer` aligned to WorldPop datalayers. This will be used as a template for producing other gridded outputs
- "adminZones" - 34 polygons in `sf` format for zonal statistics
- "clusters" - 10 small polygons in `sf` format for sample sites

Note that the adminZones and cluster boundaries are purely artificial and were created for demonstration purposes only.

For more information, see `?kampala`.

Calculations with `foot`

The core functions of `foot` are provided by `calculate_footstats()`. The operations include: 1) calculating geometry measures for each footprint, 2) summarising one or more geometry measures of footprints within zones. The simplest usage involves supplying a set of building footprint polygons and the desired characteristics to calculate. All operations return the same format - a `data.table`, optionally with a column for the index and the named column for the summarised footprint metric(s).

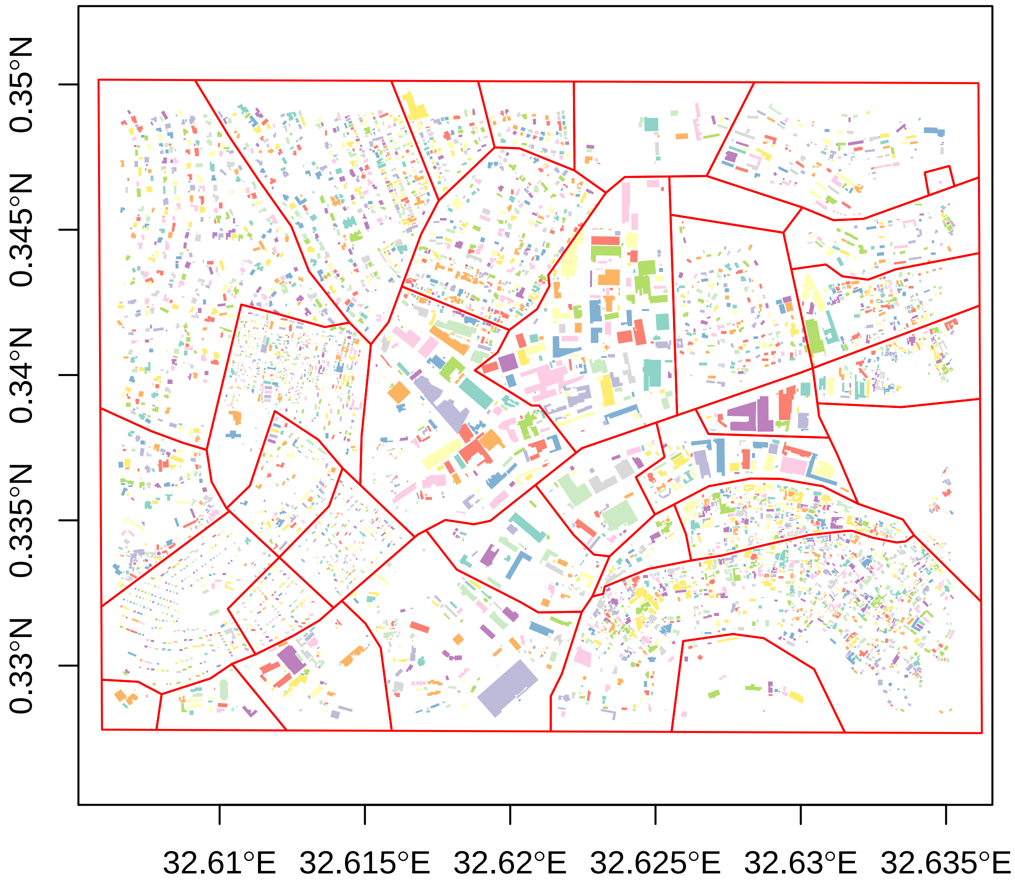

Figure 1: Sample buildings and zones in Kampala

```r
# the area and perimeter of each building footprint
built_area <- calculate_footstats(buildings, what = list("area", "perimeter"))
#> Selecting metrics
#> Setting control values.
#> Pre-calculating areas
#> Pre-calculating perimeters
#> No summary functions found, returning metrics.

head(built_area)
#>                area    perimeter
#> 1:  22.00824 [m^2] 19.26045 [m]
#> 2: 220.39011 [m^2] 67.95023 [m]
#> 3:  38.95750 [m^2] 25.37269 [m]
#> 4: 386.74429 [m^2] 98.55654 [m]
#> 5: 349.57765 [m^2] 82.22555 [m]
#> 6: 164.00931 [m^2] 55.87172 [m]
```

To summarise the footprint characteristics over all building polygons, the name of a summary function, or a list of multiple functions can be supplied.

```r
# the average and standard deviation of all building areas
calculate_footstats(buildings, what = "area", how = list("mean", "sd"))
#> Selecting metrics
#> Setting control values.
#> Pre-calculating areas
#>
#> Calculating  2  metrics ...
#>    area mean
#>    area sd
#> Finished calculating metrics.
#>    zoneID      area_mean area_sd
#> 1:      1 263.1865 [m^2]  715.28
```

Available metrics and summary measures

Currently implemented are measures for:

- building presence
- area
- perimeter
- nearest neighbour distance
- angle of rotation
- compactness and shape

A table of metrics and other packaged summary function names available for each is available using `list_fs()`. The results of this function provide "cols" and "funs" which can be passed as `what` and `how` arguments, respectively, to `calculate_footstats`.

```r
# get all available built-in functions for perimeter calculations
argsList <- list_fs(what = "perimeter")

calculate_footstats(buildings, what = argsList$cols, how = argsList$funs)
#> Selecting metrics
#> Setting control values.
#> Pre-calculating perimeters
#>
```

```
#> Calculating  7  metrics ...
#>    perimeter cv
#>    perimeter max
#>    perimeter mean
#>    perimeter median
#>    perimeter min
#>    perimeter sd
#>    perimeter sum
#> Finished calculating metrics.
#>    zoneID perimeter_cv perimeter_max perimeter_mean perimeter_median
#> 1:      1    0.9396027  1089.097 [m]   62.22027 [m]     46.68314 [m]
#>    perimeter_min perimeter_sd perimeter_sum
#> 1:  14.88332 [m]     58.46233  301146.1 [m]
```

With no other argument supplied, all the footprints are treated as coming from the same spatial zone for any summary function. A later section describes the process for identifying zones.

Additional characteristics and geometry measures

Whenever possible, `foot` makes use of generic functions within `R`. Most low-level geometry calculations rely on `sf` and `lwgeom` and users need to have recent versions of these packages installed. There are other stand-alone functions within `foot` to support more complex or less-common measurements.

Nearest neighbour distances

Distances can be calculated between footprints, or between footprints and other spatial objects. The distances can be measured edge-to-edge (`method="poly"`) or the centroids of the building footprints can be used (`method="centroid"`).

```
# nearest distance for the first 10 buildings to any other building measured
# between polygon edges
fs_nndist(buildings[1:10, ], buildings, maxSearch = 200, unit = "m")
#> Units: [m]
#>  [1]  3.961359  7.894813  1.019971 22.306277  2.484471 16.257603  0.951225
#>  [8]  1.864305  2.607113  2.291474

# omitting argument 'Y' measures distance among the footprints supplied setting
# maxSearch=NULL removes the search restriction
fs_nndist(buildings[1:10, ], method = "centroid", maxSearch = NULL)
#> Units: [m]
#>  [1]  99.95859  99.95859  90.11091  90.11091 116.64498 116.64498 217.86037
#>  [8] 228.63068 217.86037 438.91352
```

Note that distance calculations are slower for polygons and for unprojected coordinate systems. The centroid-based calculations are fast. It is recommended that a maximum search radius is always used. Internally the calculations are done with a `data.table` to benefit from multi-threading capabilities.

Rotation angles

A less conventional geometric measure is derived from the rotated bounding rectangle. This is the rectangle enclosing a footprint polygon which has been rotated to minimise the area. In contrast, a "bounding box" for a polygon is always oriented along the x and y axes.

```
# To obtain the rotated rectangle as a spatial object
mbr <- fs_mbr(buildings[4502, ], returnShape = T)
```

```r
plot(sf::st_geometry(buildings[4502, ]), col = "grey")
plot(mbr, border = "red", lwd = 2, add = T)
```

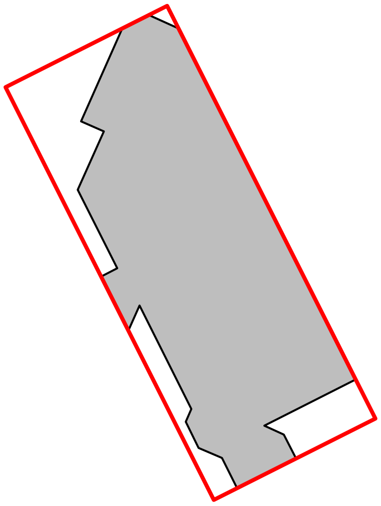

```r
# Or obtain just the angle measure
fs_mbr(buildings[4502, ], returnShape = F)
#>        X
#> 63.25363
```

The angles can be summarised as an entropy measure and normalised to describe how much the angles of a set of structures depart from a regular grid pattern (available in `calculate_footstats` where `how="entropy"`).

Creating and supplying zone indices

Rather than treating all footprints features as belonging to a single summary zone, it's more common to want to summarise metrics within smaller areas. There are several ways to supply information on the zones.

Index by vector

A vector of indices for the zones can be supplied to `foot` functions as a 1) column name within the footprints, 2) a standalone numeric vector of indices, or 3) a spatial polygons object to join to the building footprints. The length of a vector of indices must match the number of building polygons.

```r
# create a vector of ten random zones
idx <- sample(1:10, nrow(buildings), replace = T)
buildings$id <- idx  # add it to the buildings object
table(buildings$id)  # splitting observations into 10 groups
#>
```

```r
#>   1   2   3   4   5   6   7   8   9  10
#> 459 475 494 520 523 496 456 495 461 461

# 1. pass the index by name
colnames(buildings)
#> [1] "FID_1"    "geometry" "id"
calculate_footstats(buildings, "id", what = "area", how = "mean", verbose = FALSE)
#>     id      area_mean
#>  1:  1 268.4276 [m^2]
#>  2:  2 287.4527 [m^2]
#>  3:  3 277.2709 [m^2]
#>  4:  4 288.0874 [m^2]
#>  5:  5 262.0502 [m^2]
#>  6:  6 276.8436 [m^2]
#>  7:  7 239.8205 [m^2]
#>  8:  8 225.1204 [m^2]
#>  9:  9 224.6298 [m^2]
#> 10: 10 278.9229 [m^2]

# 2. pass the index as a standalone vector
calculate_footstats(buildings, idx, what = "settled", how = "count", verbose = FALSE)
#>     zoneID settled_count
#>  1:      1           459
#>  2:      2           475
#>  3:      3           494
#>  4:      4           520
#>  5:      5           523
#>  6:      6           496
#>  7:      7           456
#>  8:      8           495
#>  9:      9           461
#> 10:     10           461

# 3. pass a separate spatial object of zones
calculate_footstats(buildings, zone = adminzones, what = "angle", how = "entropy",
    verbose = FALSE)
#>     zoneID angle_entropy
#>  1:      1     0.9351095
#>  2:      2     0.3108829
#>  3:      3     0.5849276
#>  4:      4     0.2003194
#>  5:      5     0.6678707
#>  6:      6     0.7139565
#>  7:      7     0.9691374
#>  8:      8     0.6102641
#>  9:      9     0.5110688
#> 10:     10     0.7309444
#> 11:     11     0.3933987
#> 12:     12     0.4602628
#> 13:     13     1.0000000
#> 14:     14     0.4656334
#> 15:     15     0.3129308
#> 16:     16     0.4551733
```

```
#> 17:     17     0.3864071
#> 18:     18     0.6949125
#> 19:     19     0.2928137
#> 20:     20     0.4598566
#> 21:     21     0.6047045
#> 22:     22     0.6520860
#> 23:     23     0.2425743
#> 24:     24     0.4518750
#> 25:     25     0.8426361
#> 26:     26     0.7302834
#> 27:     27     0.4325040
#> 28:     29     0.6144511
#> 29:     30     0.4754793
#> 30:     31     0.6023774
#> 31:     32     0.4508777
#> 32:     33     0.8639418
#> 33:     34     0.1613791
#>     zoneID angle_entropy
```

Index by zone shapes

Rather than supplying a pre-calculated column or vector of zonal indices, buildings can be assigned a zone based on a spatial join. When the index is created in the building footprints, it will be named "zoneID" or a user-specified name.

```
# examine the other objects loaded from supplied data
head(adminzones)
#> Simple feature collection with 6 features and 1 field
#> geometry type:  POLYGON
#> dimension:      XY
#> bbox:           xmin: 32.60589 ymin: 0.3277355 xmax: 32.62531 ymax: 0.3388665
#> geographic CRS: WGS 84
#>   Id                       geometry
#> 1  1 POLYGON ((32.61231 0.327771...
#> 2  2 POLYGON ((32.61034 0.335317...
#> 3  3 POLYGON ((32.61592 0.327757...
#> 4  4 POLYGON ((32.6214 0.3277355...
#> 5  5 POLYGON ((32.62342 0.333760...
#> 6  6 POLYGON ((32.62498 0.335199...
head(clusters)
#> Simple feature collection with 6 features and 1 field
#> geometry type:  POLYGON
#> dimension:      XY
#> bbox:           xmin: 32.60624 ymin: 0.3303863 xmax: 32.63375 ymax: 0.3472696
#> geographic CRS: WGS 84
#>   Id                       geometry
#> 1  1 POLYGON ((32.6066 0.3398617...
#> 2  2 POLYGON ((32.61151 0.337602...
#> 3  3 POLYGON ((32.61028 0.331954...
#> 4  4 POLYGON ((32.61649 0.344649...
#> 5  5 POLYGON ((32.62552 0.345516...
#> 6  6 POLYGON ((32.63225 0.340927...

# Return a table of index values based on administrative zone polygons using the
```

```r
# standalone function within `foot`
zID <- zonalIndex(buildings, adminzones, returnObject = F)
head(zID, 10)  # the xID column are row numbers to object X
#>      xID zoneID
#>  1:  355      1
#>  2:  493      1
#>  3:  802      1
#>  4:  845      1
#>  5:  898      1
#>  6: 1321      1
#>  7: 1395      1
#>  8: 1551      1
#>  9: 1893      1
#> 10: 2299      1

# Alternatively (and preferably), join zones to create a new footprint object A
# custom zone name can be used but must be specificed to the summary functions
zObj <- zonalIndex(buildings, clusters, zoneField = "Id", returnObject = T)
zObj
#> Simple feature collection with 348 features and 3 fields
#> geometry type:  POLYGON
#> dimension:      XY
#> bbox:           xmin: 32.60631 ymin: 0.328119 xmax: 32.63543 ymax: 0.349785
#> geographic CRS: WGS 84
#> First 10 features:
#>     FID_1 id Id                       geometry
#> 1   53393  1  1 POLYGON ((32.60779 0.339691...
#> 2  346362  1  1 POLYGON ((32.60787 0.33845,...
#> 3   17706  2  1 POLYGON ((32.60664 0.339347...
#> 4   29106  2  1 POLYGON ((32.60719 0.339276...
#> 5  150833  2  1 POLYGON ((32.60723 0.338889...
#> 6  238291  2  1 POLYGON ((32.6073 0.339043,...
#> 7  379094  2  1 POLYGON ((32.60678 0.339284...
#> 8  101340  3  1 POLYGON ((32.6071 0.338821,...
#> 9  361577  3  1 POLYGON ((32.60825 0.339422...
#> 10  59496  4  1 POLYGON ((32.60692 0.339387...
```

When using a new spatial object which has been joined to its zones, remember to supply the name of the zone field to `calculate_foostats`.

```r
# use the new object and zone field 'Id' in a summary calculation
colnames(zObj)
#> [1] "FID_1"    "id"       "Id"       "geometry"

zarea <- calculate_footstats(zObj, zone = "Id", what = "area", how = "mean", verbose = F)
clusters <- merge(clusters, zarea, by = "Id")
plot(sf::st_geometry(adminzones))
plot(clusters["area_mean"], add = T)
```

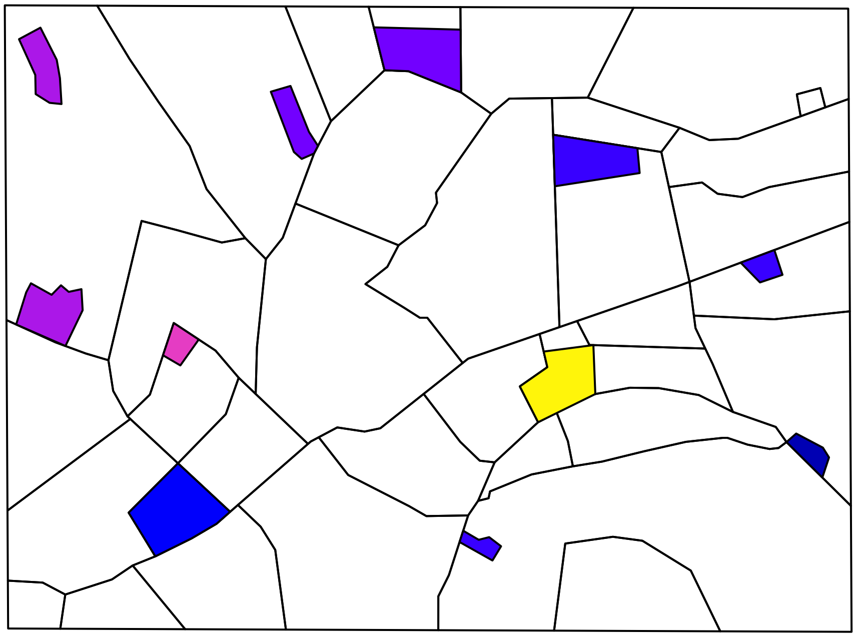

The `zonalIndex` function works by spatial intersection. This produces some (potentially useful) side effects that users need to be aware of. Specifically, note that if a building footprint intersects more than 1 zone it will be duplicated and associated to all intersecting zones.

The default behaviour (see `method`) is to assign a building to a zone based on its **centroid**.

```
# Note the selected structures extend beyond the cluster boundary
plot(sf::st_geometry(clusters)[[6]])
plot(sf::st_geometry(buildings), add = T)
plot(sf::st_geometry(zObj[zObj$Id == 6, ]), col = "red", add = T)
plot(sf::st_geometry(sf::st_centroid(zObj[zObj$Id == 6, ])), pch = 16, add = T)
```

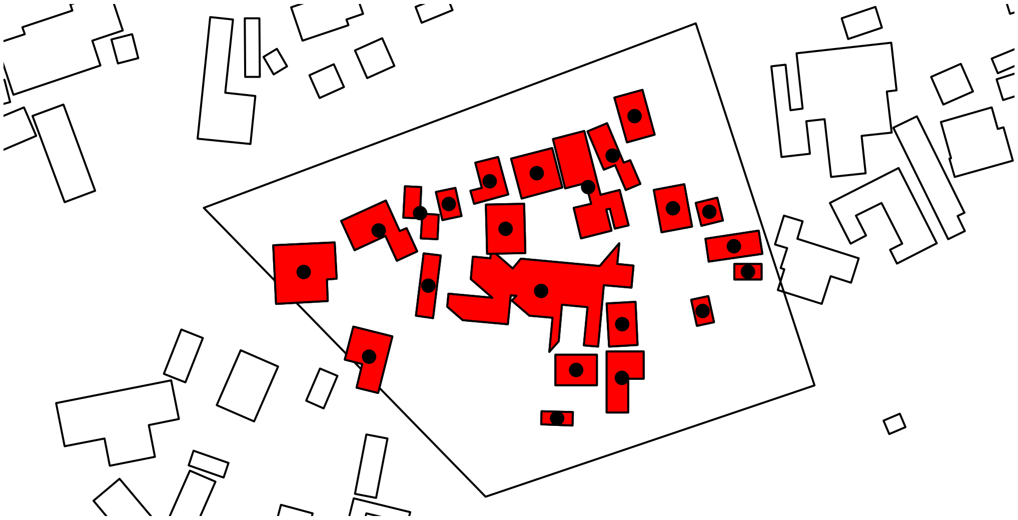

Alternatively, an intersection can be used to assign footprints to any zones which are intersected. The whole footprint is associated, even if the shape is not "contained" by the zone.

```
# Note the selected structures extend beyond the cluster boundary
zInt <- zonalIndex(buildings, clusters, zoneField = "Id", method = "intersect")

plot(sf::st_geometry(clusters)[[6]])
plot(sf::st_geometry(buildings), add = T)
plot(sf::st_geometry(zInt[zInt$Id == 6, ]), col = "red", add = T)
```

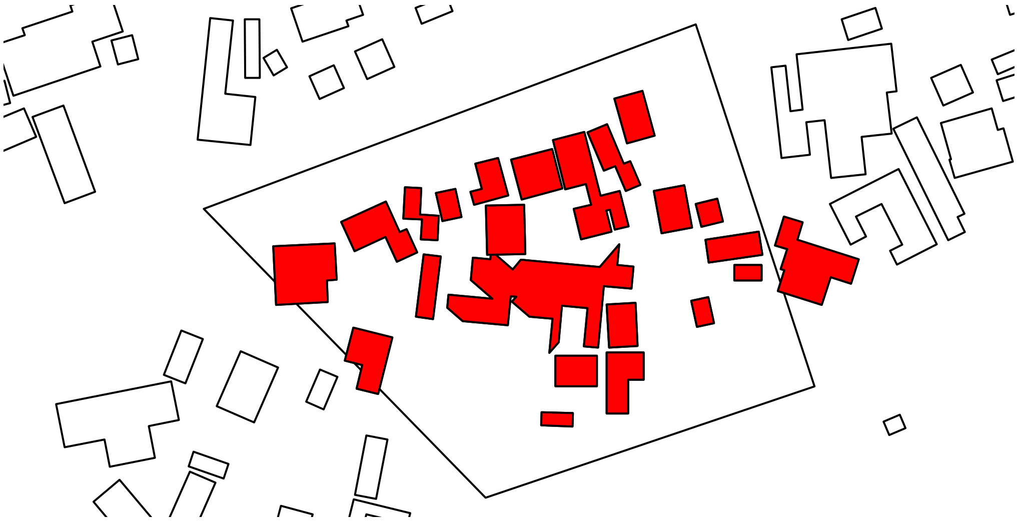

Finally, the intersection can return a clipped set of buildings.

```
zClip <- zonalIndex(buildings, clusters, zoneField = "Id", method = "clip")

plot(sf::st_geometry(clusters)[[6]])
plot(sf::st_geometry(buildings), add = T)
plot(sf::st_geometry(zClip[zClip$Id == 6, ]), col = "red", add = T)
```

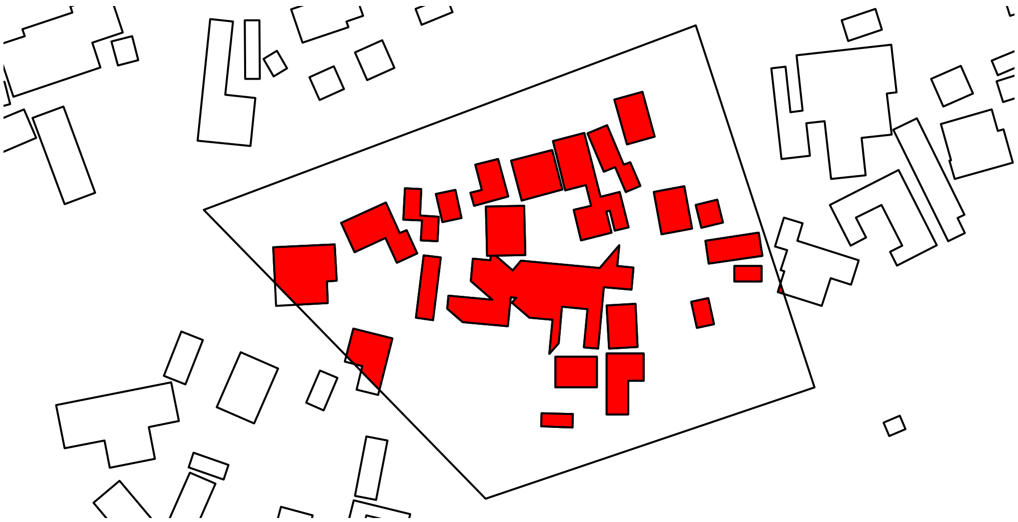

This third option clips the footprints via intersection, potentially leaving small slivers of structures in the zone which will affect the feature statistics.

An additional side effect of the intersection operation is that overlapping zones are allowed, and this can duplicate and associate footprints into both (overlapping) zones.

```
# create a temporary shape by shifting one cluster
newClusters <- st_sfc(sf::st_geometry(clusters)[[1]], sf::st_cast(sf::st_geometry(clusters)[[1]] +
    c(0.001, 1e-04), "POLYGON"), crs = sf::st_crs(clusters))

newClusters <- st_sf(geometry = newClusters, crs = sf::st_crs(clusters))

newObj <- zonalIndex(buildings, newClusters, method = "clip")

# areas of overlap are in a purple hue
plot(sf::st_geometry(newClusters))
plot(sf::st_geometry(newObj[newObj$zoneID == 1, ]), col = "red", add = T)
plot(sf::st_geometry(newObj[newObj$zoneID == 2, ]), col = sf::sf.colors(n = 1, alpha = 0.5),
    add = T)
plot(sf::st_geometry(buildings), add = T)
```

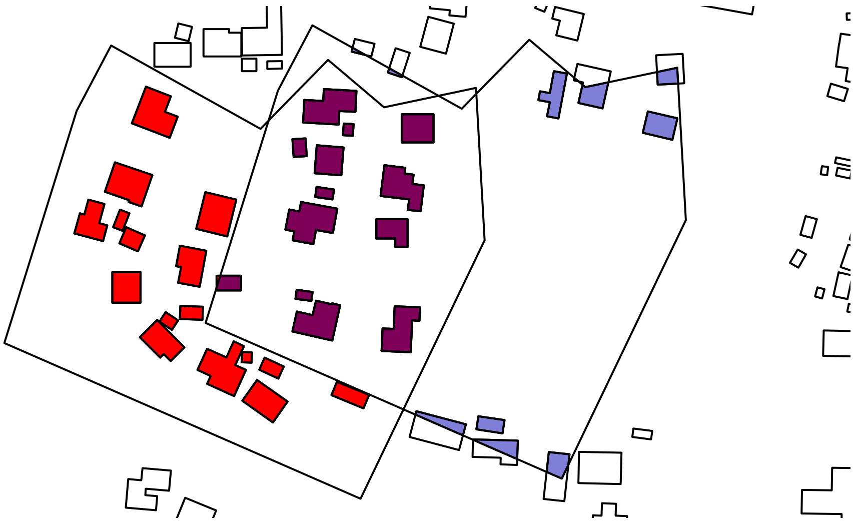

These side effects are allowed because they allow for flexibility to support types of "focal" summaries of statistics and to produce a true gridded measure of footprint metrics.

Calculating multiple metrics

The `calculate_footstats()` function provides a convenient wrapper to the individual footprint statistics and as well to `zonalIndex`. The function accepts a variety of input formats (see `?calculate_footstats`). Multiple metrics can be calculated for the same sets of buildings and zones.

```
# Creates a zonal index and calculates multiple metrics
# Use the intersection method define zones
results <- calculate_footstats(buildings,
                               zone=adminzones,
                               what="area",
                               how=list("mean","cv"),
                               controlZone=list(method="intersect"),
                               verbose=F
                              )
  results
#>      zoneID        area_mean   area_cv
#>   1:      1  402.59838 [m^2] 1.2479809
#>   2:      2  211.05341 [m^2] 1.1069491
#>   3:      3  525.07469 [m^2] 1.9194754
#>   4:      4  555.09314 [m^2] 3.8206699
#>   5:      5  568.71544 [m^2] 1.7000587
#>   6:      6 1021.95294 [m^2] 2.0652377
#>   7:      7 2027.70402 [m^2] 1.4077033
```

```
#>  8:      8  908.24803 [m^2] 1.4225521
#>  9:      9  181.68220 [m^2] 1.6104922
#> 10:     10  251.99721 [m^2] 1.5272718
#> 11:     11  220.11253 [m^2] 1.4300230
#> 12:     12   87.83690 [m^2] 1.2659928
#> 13:     13  661.61890 [m^2] 1.7191007
#> 14:     14   81.85444 [m^2] 0.3447115
#> 15:     15   80.78532 [m^2] 1.0115881
#> 16:     16   68.75161 [m^2] 0.5102143
#> 17:     17  139.17524 [m^2] 0.8678702
#> 18:     18   87.23691 [m^2] 1.5248900
#> 19:     19  237.07627 [m^2] 0.6189696
#> 20:     20  199.39362 [m^2] 0.9103598
#> 21:     21  244.09906 [m^2] 2.4147823
#> 22:     22  192.39558 [m^2] 0.3464272
#> 23:     23  776.00000 [m^2] 2.1447695
#> 24:     24  219.27229 [m^2] 0.7972001
#> 25:     25  506.22155 [m^2] 1.3228666
#> 26:     26 1129.64270 [m^2] 1.7076343
#> 27:     27  228.00865 [m^2] 2.2623265
#> 28:     29  238.76025 [m^2] 2.7887959
#> 29:     30  203.68136 [m^2] 0.8303153
#> 30:     31  142.13649 [m^2]        NA
#> 31:     32  272.76984 [m^2] 1.0894764
#> 32:     33  418.66391 [m^2] 0.9787568
#> 33:     34  176.81732 [m^2] 1.3707628
#>     zoneID        area_mean   area_cv
```

Multiple metrics can be applied to specific groups of characteristics by providing nested lists of metrics and summary functions. The argument `what` will accept a string or a list of strings for specific metric names. Users may also supply `"all"` or `"nodist"` to calculate all available metrics or all bar the nearest neighbour distance-related ones, respectively. Excluding the distance-related metrics can speed up the calculations due to the long-running. Other performance improvements can be to set `controlDistance=list("method"="centroid")`, which uses centroid-to-centroid nearest neighbour distances rather than polygon edge-to-edge. See also, `?fs_nndist`.

```
# Use nested lists to group characteristics and summary functions
results <- calculate_footstats(buildings,
                               zone=adminzones,
                               what=list(list("area","perimeter"),
                                         list("settled")),
                               how=list(list("sum","cv"),
                                        list("count")),
                               controlZone=list(method="centroid"),
                               verbose=F
                              )
  results
#>     zoneID          area_sum   perimeter_sum   area_cv perimeter_cv
#>  1:      1   8454.5661 [m^2]  1781.62245 [m] 1.2479809    0.6561304
#>  2:      2  29758.5314 [m^2]  8596.30974 [m] 1.1069491    0.7313348
#>  3:      3  25728.6600 [m^2]  4414.86934 [m] 1.9194754    0.9811076
#>  4:      4  63835.7113 [m^2]  9640.94681 [m] 3.8206699    1.0600954
#>  5:      5  26729.6259 [m^2]  4329.10334 [m] 1.7000587    0.9581201
#>  6:      6  35768.3531 [m^2]  3835.74843 [m] 2.0652377    0.9616081
```

```
#>  7:      7  28387.8563 [m^2]  2565.09927 [m] 1.4077033    0.9286094
#>  8:      8  44504.1537 [m^2]  6348.37632 [m] 1.4225521    0.8489816
#>  9:      9  48145.7825 [m^2] 15739.78807 [m] 1.6104922    0.9246860
#> 10:     10   7559.9164 [m^2]  2087.88260 [m] 1.5272718    1.0900293
#> 11:     11  24212.3780 [m^2]  7672.66021 [m] 1.4300230    0.8815988
#> 12:     12   3109.8042 [m^2]  1403.25960 [m] 1.3014215    0.7257624
#> 13:     13   2646.4756 [m^2]   423.87801 [m] 1.7191007    1.3165558
#> 14:     14   6712.0641 [m^2]  3088.40482 [m] 0.3447115    0.2059276
#> 15:     15  19226.9062 [m^2]  8676.96497 [m] 1.0115881    0.5073846
#> 16:     16   9900.2318 [m^2]  4968.39946 [m] 0.5102143    0.3095971
#> 17:     17  14752.5754 [m^2]  5213.18182 [m] 0.8678702    0.5541695
#> 18:     18  36895.4355 [m^2] 15742.13708 [m] 1.5308486    0.7093738
#> 19:     19  96490.0399 [m^2] 26354.10548 [m] 0.6189696    0.4151437
#> 20:     20  80355.6287 [m^2] 23566.14546 [m] 0.9103598    0.5257108
#> 21:     21  18063.3304 [m^2]  4403.44976 [m] 2.4147823    0.7810948
#> 22:     22  10196.9657 [m^2]  3061.47987 [m] 0.3464272    0.2238327
#> 23:     23 132673.0618 [m^2] 17464.78357 [m] 2.1625263    1.1205392
#> 24:     24  61834.7854 [m^2] 17707.47705 [m] 0.7972001    0.5317242
#> 25:     25  11643.0956 [m^2]  2163.70761 [m] 1.3228666    0.8940386
#> 26:     26 150208.7190 [m^2] 17604.40321 [m] 1.6814458    1.0767096
#> 27:     27  33816.8355 [m^2] 10017.95165 [m] 0.7963207    0.4462858
#> 28:     29  37007.8380 [m^2]  9185.83050 [m] 2.7887959    1.0713172
#> 29:     30  15276.1021 [m^2]  4621.61764 [m] 0.8303153    0.5426390
#> 30:     31    142.1365 [m^2]    48.13661 [m]        NA           NA
#> 31:     32  35732.8487 [m^2]  9127.30909 [m] 1.0894764    0.6469339
#> 32:     33   7117.2864 [m^2]  1557.24261 [m] 0.9787568    0.6104610
#> 33:     34 146935.1905 [m^2] 47733.83601 [m] 1.3707628    0.7474269
#>     zoneID          area_sum   perimeter_sum   area_cv perimeter_cv
#>     settled_count
#>  1:            21
#>  2:           141
#>  3:            49
#>  4:           115
#>  5:            47
#>  6:            35
#>  7:            14
#>  8:            49
#>  9:           265
#> 10:            30
#> 11:           110
#> 12:            36
#> 13:             4
#> 14:            82
#> 15:           238
#> 16:           144
#> 17:           106
#> 18:           424
#> 19:           407
#> 20:           403
#> 21:            74
#> 22:            53
#> 23:           172
#> 24:           282
```

```
#> 25:            23
#> 26:           129
#> 27:           177
#> 28:           155
#> 29:            75
#> 30:             1
#> 31:           131
#> 32:            17
#> 33:           831
#>     settled_count
```

Filtering buildings

In some settings it may be preferable to exclude very small and/or very large building footprint polygons. The lower and upper bounds for filtering can be specified with `minArea` and `maxArea` in the `filter` argument. The values for these filters are in the same units specified by `controlUnits` or the default value for area calculations. Note that an "area" footprint statistic does not need to be requested as this characteristic is automatically calculated to enable filtering.

```
# Filtering: # footprints must be larger than 50 m^2 and smaller than 1000 m^2
calculate_footstats(buildings,
                    what="perimeter",
                    how=list("mean", "sum"),
                    controlUnits=list(areaUnit="m^2"),
                    filter=list(minArea=50, maxArea=1000),
                    verbose=FALSE)
#>    zoneID perimeter_mean perimeter_sum
#> 1:      1   63.16899 [m]  228482.2 [m]
```

Next steps

The `calculate_footstats` function provides the core functionality for calculating and summarising the characteristics of building footprints. It also wraps the functionality of assigning footprints to zones based on different spatial joining techniques. To go further with `foot` the concept of footprint morphology calculations can be extended to created gridded data. See `vignette("bigfoot", package="foot")`. Additionally, users can specify their own custom summary functions to be used with `calculate_footstats`. This and other advanced options are covered in `vignette("cobbler", package="foot")`.

```
sessionInfo()
#> R version 3.6.3 (2020-02-29)
#> Platform: x86_64-pc-linux-gnu (64-bit)
#> Running under: Ubuntu 20.04.1 LTS
#>
#> Matrix products: default
#> BLAS:   /usr/lib/x86_64-linux-gnu/blas/libblas.so.3.9.0
#> LAPACK: /usr/lib/x86_64-linux-gnu/lapack/liblapack.so.3.9.0
#>
#> locale:
#>  [1] LC_CTYPE=en_GB.UTF-8       LC_NUMERIC=C
#>  [3] LC_TIME=en_GB.UTF-8        LC_COLLATE=en_GB.UTF-8
#>  [5] LC_MONETARY=en_GB.UTF-8    LC_MESSAGES=en_GB.UTF-8
#>  [7] LC_PAPER=en_GB.UTF-8       LC_NAME=C
#>  [9] LC_ADDRESS=C               LC_TELEPHONE=C
```

```
#> [11] LC_MEASUREMENT=en_GB.UTF-8 LC_IDENTIFICATION=C
#>
#> attached base packages:
#> [1] stats     graphics  grDevices utils     datasets  methods   base
#>
#> other attached packages:
#> [1] sf_0.9-6 foot_0.6
#>
#> loaded via a namespace (and not attached):
#>  [1] Rcpp_1.0.5         highr_0.8          pillar_1.4.3       compiler_3.6.3
#>  [5] formatR_1.7        class_7.3-16       iterators_1.0.13   tools_3.6.3
#>  [9] digest_0.6.27      evaluate_0.14      lifecycle_0.2.0    tibble_3.0.1
#> [13] pkgconfig_2.0.3    rlang_0.4.8        foreach_1.5.1      DBI_1.1.0
#> [17] filelock_1.0.2     yaml_2.2.1         parallel_3.6.3     xfun_0.18
#> [21] e1071_1.7-4        stringr_1.4.0      dplyr_1.0.2        knitr_1.30
#> [25] generics_0.0.2     vctrs_0.3.4        tidyselect_1.1.0   classInt_0.4-3
#> [29] grid_3.6.3         glue_1.4.2         data.table_1.13.2  R6_2.5.0
#> [33] rmarkdown_2.1      purrr_0.3.4        magrittr_1.5       stars_0.4-3
#> [37] codetools_0.2-16   htmltools_0.4.0    ellipsis_0.3.1     units_0.6-7
#> [41] abind_1.4-5        KernSmooth_2.23-16 stringi_1.4.6      doParallel_1.0.16
#> [45] lwgeom_0.2-5       crayon_1.3.4
```

Gridded footprint calculation layers

December 10, 2020

The `foot` package was developed by WorldPop at the University of Southampton (www.worldpop.org) to support geometric calculations and zonal summaries of measures from building footprint polygons. The `vignette("footsteps", package="foot")` provides an introduction to package and the functionality of calculating and summarising morphology measures. This vignette builds on those methods and demonstrates a more advanced workflow to produce gridded summaries of buildings measures. In particular, the focus is on using `foot::calculate_bigfoot()` in order how to handle very large (i.e. national-scale) datasets of building footprints.

```
library(foot)
```

Calculations with `foot`

The central function for producing gridded layers is `calculate_bigfoot`. It is designed to support processing large sets of building polygons into gridded (GeoTiff) summary layers. The procedure is intended to be more memory-efficient by splitting the processing area into "tiles" which can then be handled individually or in parallel. This function works as a wrapper to `calculate_footstats` as well as several helper functions within the `foot` package for managing the input/output and creating and indexing spatial zones. Because it extends `calculate_footstats`, this function takes the same `what=` and `how=` arguments to define the summary operations. Likewise the available metrics are available from `list_fs()`.

By default the function performs calculations in parallel (which can be changed with the argument `parallel=FALSE`). Users can adjust the number of computing cores used with the argument `nCores=`) To monitor the supplied values and processing steps, set `verbose=TRUE`.

Main inputs

Users need to supply `calculate_bigfoot()` with:

- A path to a file of building footprints in a spatial vector data format (e.g. `.gpkg`, `.shp`)
- A filepath to a template gridded dataset specifying the extent and resolution for the outputs (e.g. `.tif`)

For example:

While `R` objects can be supplied for the function parameters (as will be shown in this example), it is recommended to supply character strings of paths to the files. `calculate_bigfoot` will only read the subset of data needed for a particular processing step. This is intended to allow users to process much larger datasets than can be held in memory.

Basic calculations

With the key inputs for the file paths set, the processing can be executed with a single function.

```
# basic function call with default parameters
calculate_bigfoot(X=buildings,
                  template=mgrid,
                  what="settled", how="binary",
                  parallel=FALSE, verbose=TRUE)
#> trying to read file: C:\Users\Admin\Documents\GitHub\foot\wd\in\kampala_grid.tif
```

```
#> Selecting metrics
#> Setting control values
#> Creating output grids
#> Creating list of processing tiles
#>
#> Tile: 1 of 1
#> Reading footprints
#> Reading template grid
#> Selecting metrics
#> Setting control values.
#> Creating zonal index
#>
#> Calculating  1  metrics ...
#>    settled binary
#> Finished calculating metrics.
#> Writing output tiles
#> Finished writing grids
#>
#> Finished processing all tiles: 2020-12-10 11:50:10
```

The documentation and default parameters are available from `?foot::calculate_bigfoot`.

The arguments `what` and `how` are the same as for `calculate_footstats`. They provide a character or list list of character names or characteristics (i.e. columns within the building footprint attributes) and summary functions, respectively. The available metrics can be retrieved with `list_fs()`.

Specifying outputs

By default the outputs of `bigfoot` are saved as GeoTiffs in the user's working directory (`getwd()`). Each grid is named by the combination of characteristics and summary function. The example below shows how to retrieve an output of `calculate_bigfoot`.

```
# retrieve the gridded outputs
outGrid <- raster::raster(file.path(getwd(), "settled_binary.tif"))

raster::plot(outGrid)
```

Users can specify an output path to another folder location (`outputPath=`). Additionally a "tag" can be specified as a parameter to the function (`outputTag=`). The tag is appended to the beginning of each file name with an underscore. This can be useful for identifying different outputs. These options are demonstrated in the code block below.

```
# basic function call specifying output parameters
calculate_bigfoot(X=buildings,
                  template=mgrid,
                  what="settled", how="binary",
                  outputPath=tempdir(),
                  outputTag="TAG",
                  parallel=FALSE, verbose=TRUE)
#> trying to read file: C:\Users\Admin\Documents\GitHub\foot\wd\in\kampala_grid.tif
#> Selecting metrics
#> Setting control values
#> Creating output grids
#> Creating list of processing tiles
#>
#> Tile: 1 of 1
```

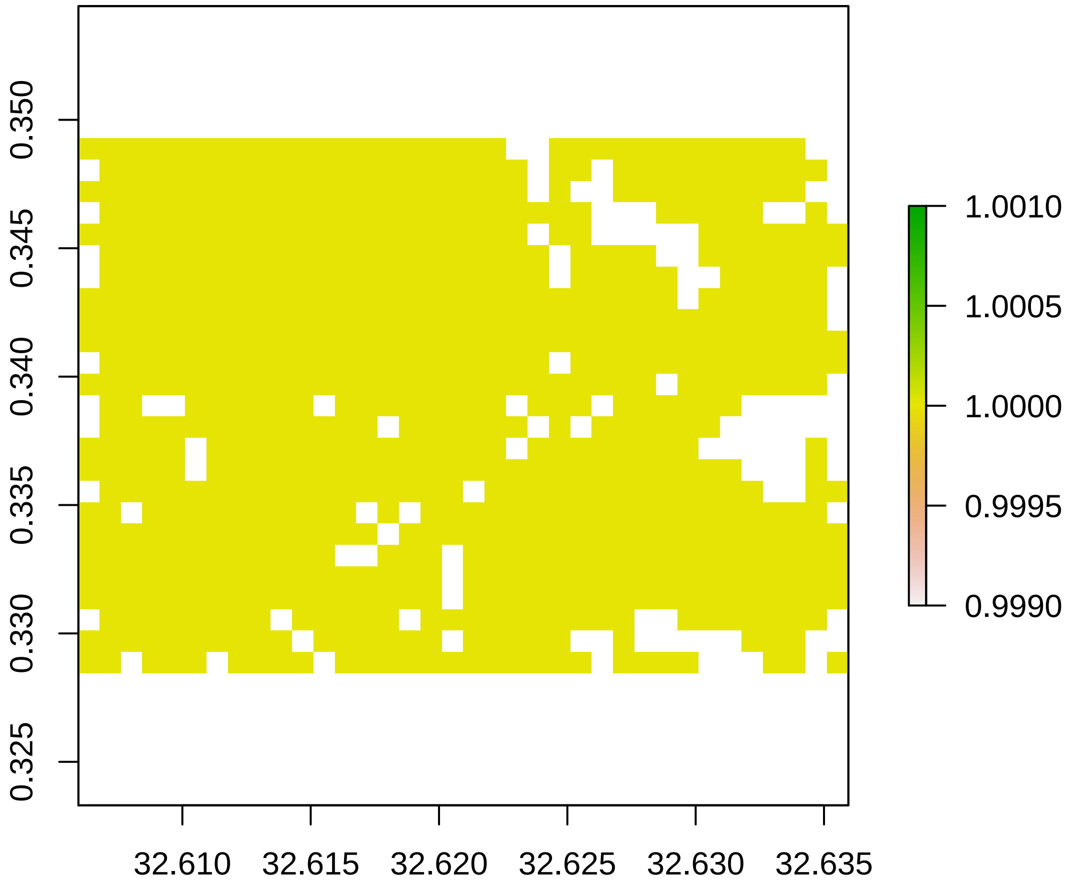

Figure 1: Binary settled raster at 100m resolution

```
#> Reading footprints
#> although coordinates are longitude/latitude, st_intersects assumes that they are planar
#> Reading template grid
#> Selecting metrics
#> Setting control values.
#> Creating zonal index
#>
#> Calculating  1  metrics ...
#>    settled binary
#> Finished calculating metrics.
#> Writing output tiles
#> Finished writing grids
#>
#> Finished processing all tiles: 2020-12-10 11:50:12
```

Multiple metrics

As with `calculate_footstats` multiple metrics and summary statistics can be supplied to `bigfoot` as a list or nested lists grouping characteristics and functions.

```
# re-running the basic call with multiple metrics
calculate_bigfoot(X=buildings,
                  template=mgrid,
                  what=list(list("settled"), list("area")),
                  how=list(list("binary"), list("cv")),
                  outputPath=tempdir(),
                  outputTag="TAG", parallel=FALSE)
#> trying to read file: C:\Users\Admin\Documents\GitHub\foot\wd\in\kampala_grid.tif
#> Selecting metrics
#> Setting control values
#> Creating output grids
#> Creating list of processing tiles
#>
#> Tile: 1 of 1
#> Reading footprints
#> Reading template grid
#> Selecting metrics
#> Setting control values.
#> Pre-calculating areas
#> Creating zonal index
#>
#> Calculating  2  metrics ...
#>    settled binary
#>    area cv
#> Finished calculating metrics.
#> Writing output tiles
#> Finished writing grids
#>
#> Finished processing all tiles: 2020-12-10 11:50:15
```

```
# retrieve the gridded outputs Note: must add the 'tag' to the filename
outGrid <- raster::raster(file.path(tempdir(), "TAG_area_cv.tif"))

raster::plot(outGrid)
```

```r
plot(sf::st_geometry(buildings), fill = NA, add = T)
```

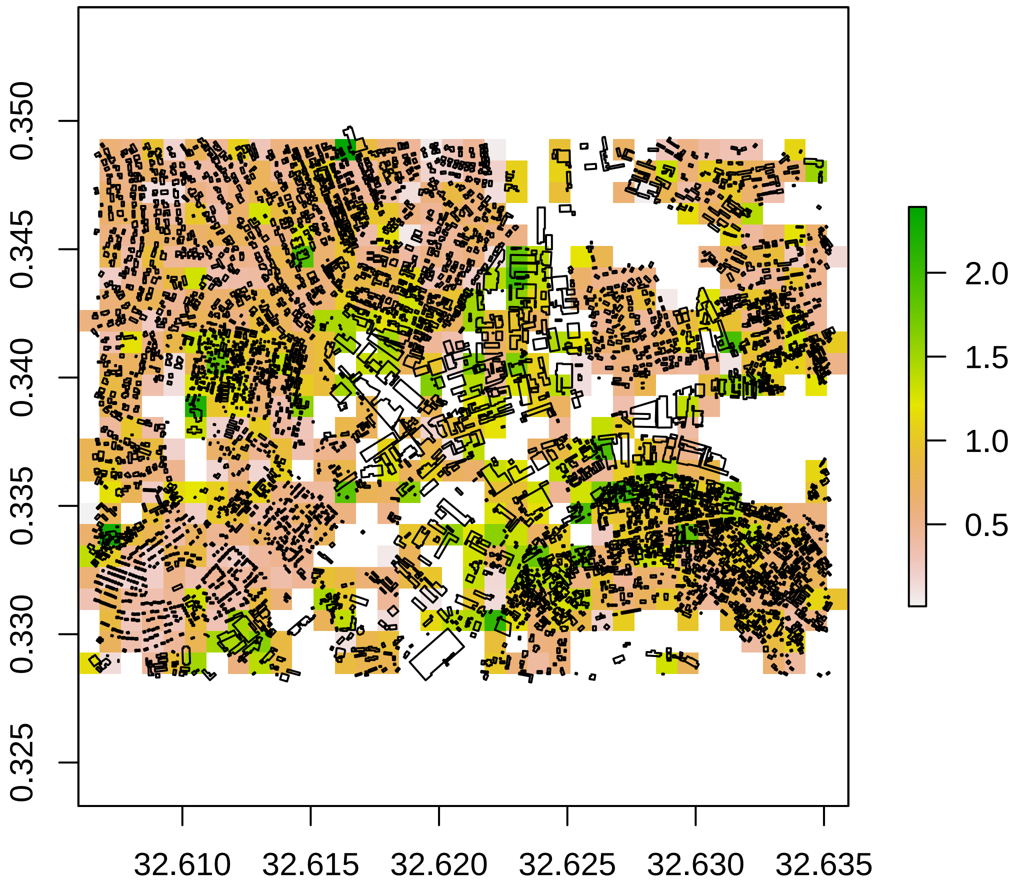

Figure 2: Coeff. of variation in building area at 100m resolution

Focal window statistics

In the examples above, the footprint statistics are summarised for buildings whose centroid intersect the pixels of the template grid. Internally this is handled by `zonalIndex()`. The method for assigning footprints to spatial zones can be adjusted using `controlZone` options.

It is also possible to extend those zones beyond the individual pixels and to calculate and summarise building footprints from within a local, circular window centred on each pixel. The output is still associated with each template grid cell. This process is similar to a moving window or filter analysis. The focal radius distance is specified in meters.

A hypothetical example of using a 300 m focal radius around a pixel centroid to construct a local zone and using

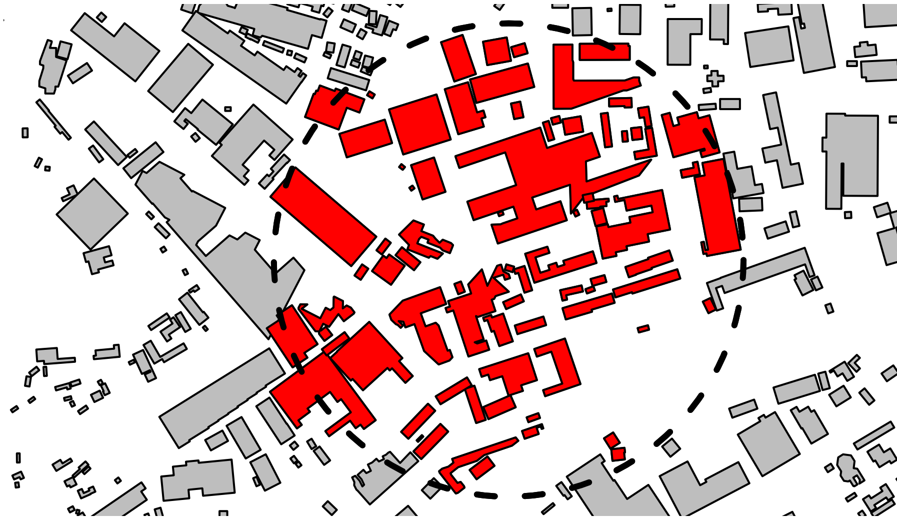

building centroids is shown below.

Below is an example of applying a 300 m focal radius in action for a gridded output layer.

```
# moving focal window calculations
calculate_bigfoot(X=buildings,
                  template=mgrid,
                  what=list(list("area"), list("perimeter")),
                  how=list(list("mean"), list("sum")),
                  focalRadius=300,  # buffer radius in meters
                  outputPath=tempdir(),
                  outputTag="TAG", parallel=FALSE)
#> trying to read file: C:\Users\Admin\Documents\GitHub\foot\wd\in\kampala_grid.tif
#> Selecting metrics
#> Setting control values
#> Creating output grids
#> Creating list of processing tiles
#>
#> Tile: 1 of 1
#> Reading footprints
#> Reading template grid
#> Buffering processing sites
#> Selecting metrics
#> Setting control values.
#> Pre-calculating areas
#> Pre-calculating perimeters
#> Creating zonal index
#>
#> Calculating  2  metrics ...
```

```
#>    area mean
#>    perimeter sum
#> Finished calculating metrics.
#> Writing output tiles
#> Finished writing grids
#>
#> Finished processing all tiles: 2020-12-10 11:50:21
```

Creating the focal window is handled by `calculate_bigfoot`. The focal window is also taken into consideration by the function when creating processing tiles - the footprints are extract from outside the tile, in neighbouring areas, to prevent edge effects

Note that when a focal radius is specified, this value is always appended to the end of the file names so that the outputs can be identified.

```
# Note that the filename includes both a tag and a focal radius value
outGrid <- raster::raster(file.path(tempdir(), "TAG_perimeter_sum_300.tif"))

raster::plot(outGrid)
```

Options and finer control

The `calculate_bigfoot` function is set up with default values that should work under most conditions; however, there is additional flexibility for users to specify alternative parameters.

Specifying geometry units

To override the default units used in the geometry calculations, a named list of unit strings can be supplied to the `controlUnits` argument. This list can contain named items for `areaUnit`, `perimUnit`, and `distUnit`. The default values are meters ("m") and square meters ("m^2") The value of each item should be coercible with `units::as_units`.

```
# change the default units used to calculate area and distance
calculate_bigfoot(X=buildings,
                  template=mgrid,
                  what=list(list("area"), list("perimeter")),
                  how=list(list("mean"), list("sum")),
                  controlZone=list("method"="intersect"), # join by intersection
                  controlUnits=list(areaUnit="m^2",  # change default units
                                    perimUnit="km"),
                  outputPath=tempdir(),
                  outputTag="TAG",
                  parallel=FALSE,
                  verbose=TRUE)
#> trying to read file: C:\Users\Admin\Documents\GitHub\foot\wd\in\kampala_grid.tif
#> Selecting metrics
#> Setting control values
#> Creating output grids
#> Creating list of processing tiles
#>
#> Tile: 1 of 1
#> Reading footprints
#> Reading template grid
#> Selecting metrics
#> Setting control values.
#> Pre-calculating areas
```

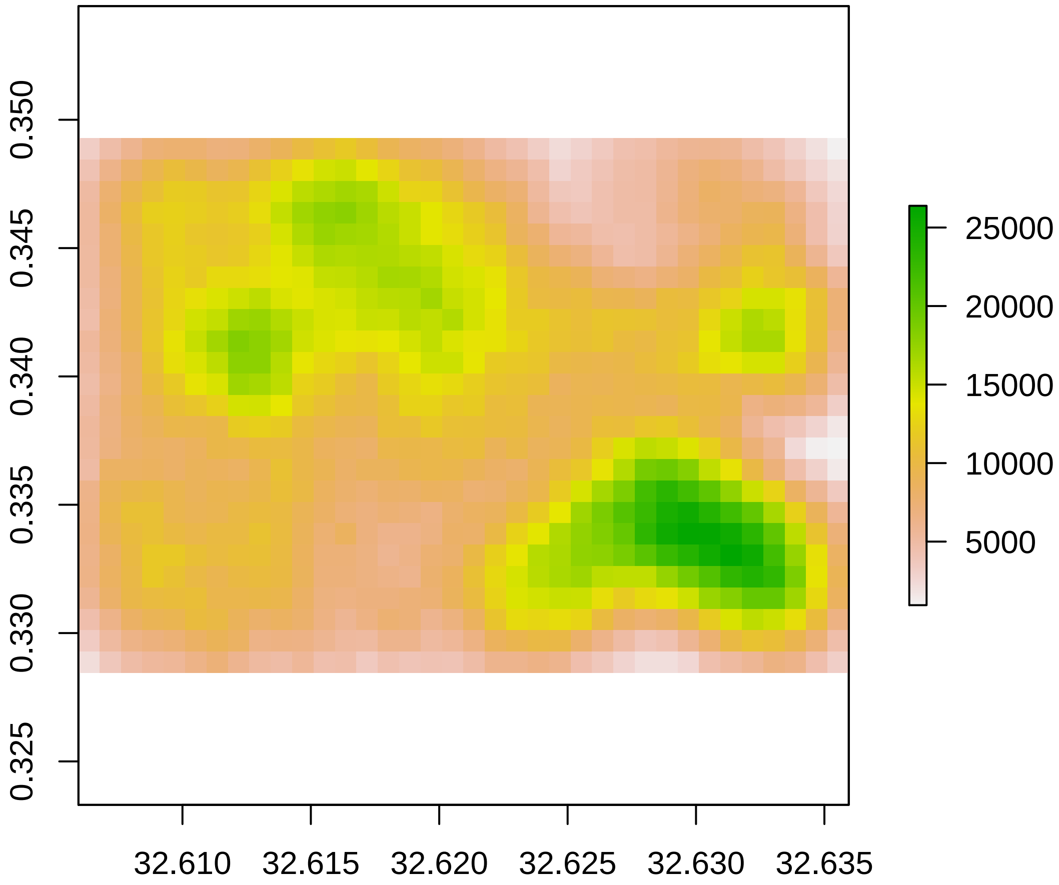

Figure 3: Total building perimeter in a 300m radius window. Output at a 100m resolution

```
#> Pre-calculating perimeters
#> Creating zonal index
#>
#> Calculating  2  metrics ...
#>    area mean
#>    perimeter sum
#> Finished calculating metrics.
#> Writing output tiles
#> Finished writing grids
#>
#> Finished processing all tiles: 2020-12-10 11:50:24

# plot the total perimeter, measured in kilometres
outGrid <- raster::raster(file.path(tempdir(), "TAG_perimeter_sum.tif"))
raster::plot(outGrid)
```

Filtering buildings

In some settings it may be preferable to exclude very small and/or very large building footprint polygons. The lower and upper bounds for filtering can be specified with `minArea` and `maxArea` in the `filter` argument. The values for these filters are in the same units specified by `controlUnits` or the default value for area calculations. Note that an "area" footprint statistic does not need to be requested as this characteristic is automatically calculated to enable filtering.

```
# Filtering: # footprints must be larger than 50 m^2 and smaller than 1000 m^2
calculate_bigfoot(X=buildings,
                  template=mgrid,
                  what=list(list("shape"), list("settled"), list("perimeter")),
                  how=list(list("mean"), list("count"), list("sum")),
                  controlUnits=list(areaUnit="m^2"),
                  filter=list(minArea=50, maxArea=1000),
                  outputPath=tempdir(),
                  parallel=FALSE,
                  verbose=TRUE)
#> trying to read file: C:\Users\Admin\Documents\GitHub\foot\wd\in\kampala_grid.tif
#> Selecting metrics
#> Setting control values
#> Creating output grids
#> Creating list of processing tiles
#>
#> Tile: 1 of 1
#> Reading footprints
#> Reading template grid
#> Selecting metrics
#> Setting control values.
#> Pre-calculating areas
#> Pre-calculating perimeters
#> Pre-calculating shape
#> Creating zonal index
#>  Filtering features larger than 50
#>  Filtering features smaller than 1000
#>
#> Calculating  3  metrics ...
#>    shape mean
```

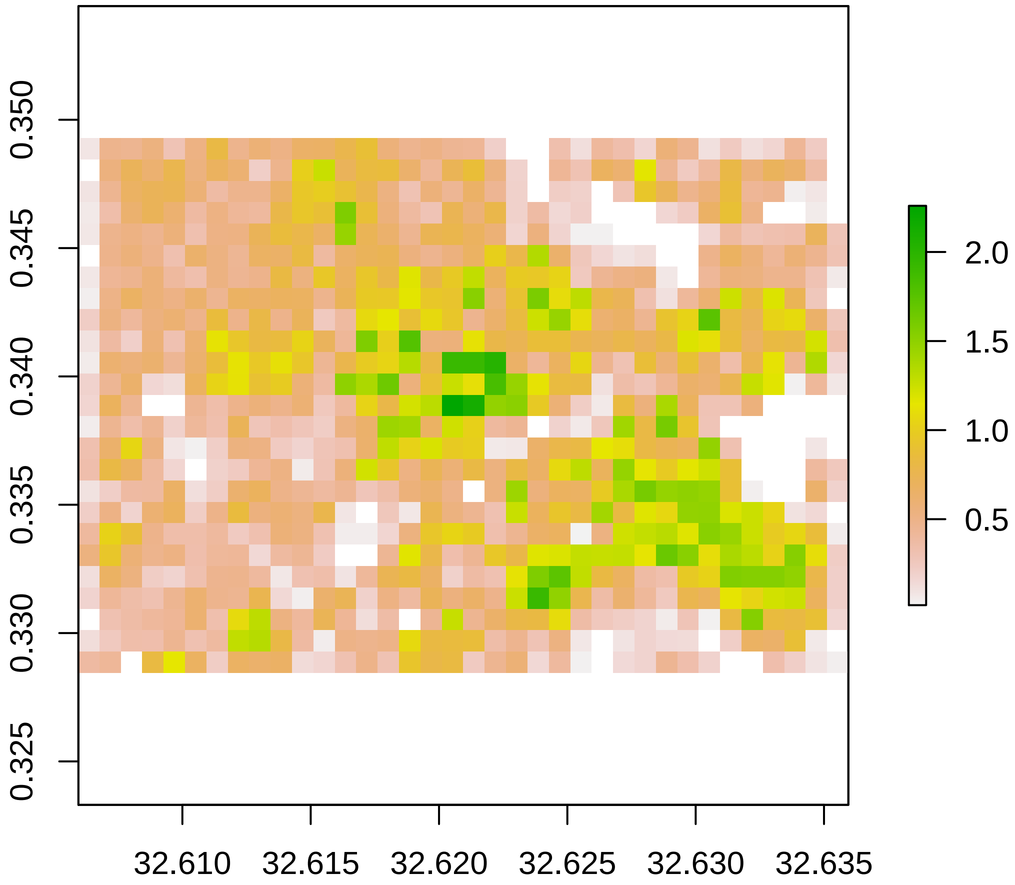

Figure 4: Total building perimeter in KM

```
#>    settled count
#>    perimeter sum
#> Finished calculating metrics.
#> Writing output tiles
#> Finished writing grids
#>
#> Finished processing all tiles: 2020-12-10 11:50:31
```

In the map of the results below, each pixel is the count of footprints present. Note the smaller number of structures and sparseness of structures in pixels around the centre portions of the image. This corresponds with the a business district and industrial areas with fewer, but larger structures.

```
outGrid <- raster::raster(file.path(tempdir(), "settled_count.tif"))

raster::plot(outGrid)
```

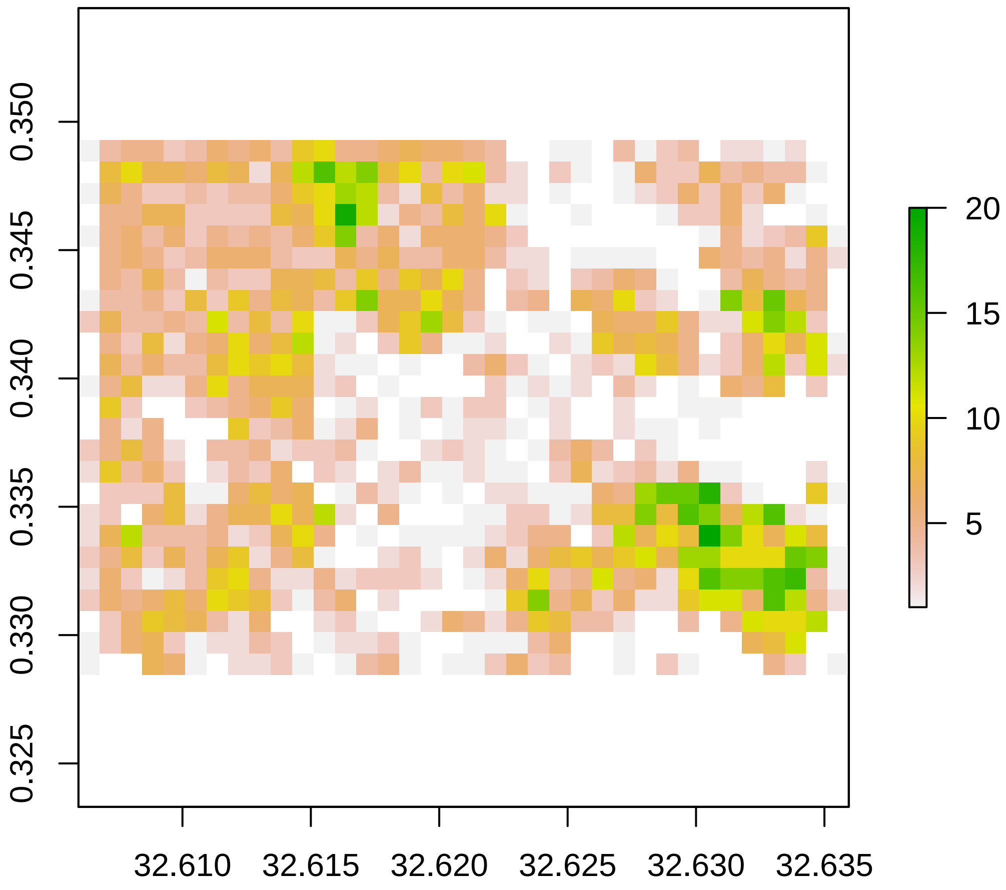

Figure 5: Count of buildings with area >50 m^2 and <1000 m^2

Tile size

The size of the processing tiles, specified in pixel dimensions (rows, columns) can be an important factor in the efficiency of the calculations. Smaller tile regions result in fewer building footprints being read/processed at one time, but there is an overhead computational cost of reading/writing files. The default value is 500 pixels. For the small demonstration shown here that results in one tile for the whole region. To show multiple tile processing, a small size is supplied and the processing is done in parallel with verbose output.

```
calculate_bigfoot(X=buildings,
                  template=mgrid,
                  what="compact",
                  how="mean",
                  tileSize=c(20, 20),  # rows x columns in pixels
                  parallel=FALSE,
                  verbose=TRUE)
#> trying to read file: C:\Users\Admin\Documents\GitHub\foot\wd\in\kampala_grid.tif
#> Selecting metrics
#> Setting control values
#> Creating output grids
#> Creating list of processing tiles
#>
#> Tile: 1 of 4
#> Reading footprints
#> Reading template grid
#> Selecting metrics
#> Setting control values.
#> Pre-calculating compactness
#> Creating zonal index
#>
#> Calculating  1  metrics ...
#>    compact mean
#> Finished calculating metrics.
#> Writing output tiles
#> Finished writing grids
#>
#> Tile: 2 of 4
#> Reading footprints
#> Reading template grid
#> Selecting metrics
#> Setting control values.
#> Pre-calculating compactness
#> Creating zonal index
#>
#> Calculating  1  metrics ...
#>    compact mean
#> Finished calculating metrics.
#> Writing output tiles
#> Finished writing grids
#>
#> Tile: 3 of 4
#> Reading footprints
#> Reading template grid
#> Selecting metrics
#> Setting control values.
#> Pre-calculating compactness
```

```
#> Creating zonal index
#>
#> Calculating  1  metrics ...
#>    compact mean
#> Finished calculating metrics.
#> Writing output tiles
#> Finished writing grids
#>
#> Tile: 4 of 4
#> Reading footprints
#> Reading template grid
#> Selecting metrics
#> Setting control values.
#> Pre-calculating compactness
#> Creating zonal index
#>
#> Calculating  1  metrics ...
#>    compact mean
#> Finished calculating metrics.
#> Writing output tiles
#> Finished writing grids
#>
#> Finished processing all tiles: 2020-12-10 11:50:37
```

Restarting

When running parallel processes on clusters, it is not currently possible to show a progress bar. This issue will hopefully be addressed in the future. However, when `verbose=TRUE`, `bigfoot` will write a log file to the output directory with the gridded data. This file is a list of tile ID numbers that have been processed. Tiles (created with `gridTiles`) are numbered sequentially from top-left to bottom-right of the template grid. On long-running jobs, the log of completed tiles can be used to monitor progress. Note that each processor will write its results as they are finished, so tiles may not be completed in order.

If a processing job crashes, the log file can also be used to restart the job. Setting the argument `restart` to a number will restart processing from that tile. Alternatively a sequence of numbers, given as a vector, can be used to re-run only certain tiles. The output grids will not be overwritten when restarting.

Next steps

This vignette has provided an overview of how to create gridded outputs layers summarising building footprint morphology measures. This workflow uses `calculate_bigfoot` and is designed to work for large sets of data through tiled read/writing and processing these tiles in parallel. The `bigfoot` functionality extends and makes use of `footstats`. Both of these functions can take use-supplied and custom summary functions. This advanced usage is demonstrated in `vignette("cobbler", package="foot")`.

```
sessionInfo()
#> R version 3.6.3 (2020-02-29)
#> Platform: x86_64-pc-linux-gnu (64-bit)
#> Running under: Ubuntu 20.04.1 LTS
#>
#> Matrix products: default
#> BLAS:   /usr/lib/x86_64-linux-gnu/blas/libblas.so.3.9.0
#> LAPACK: /usr/lib/x86_64-linux-gnu/lapack/liblapack.so.3.9.0
```

```
#>
#> locale:
#>  [1] LC_CTYPE=en_GB.UTF-8       LC_NUMERIC=C
#>  [3] LC_TIME=en_GB.UTF-8        LC_COLLATE=en_GB.UTF-8
#>  [5] LC_MONETARY=en_GB.UTF-8    LC_MESSAGES=en_GB.UTF-8
#>  [7] LC_PAPER=en_GB.UTF-8       LC_NAME=C
#>  [9] LC_ADDRESS=C               LC_TELEPHONE=C
#> [11] LC_MEASUREMENT=en_GB.UTF-8 LC_IDENTIFICATION=C
#>
#> attached base packages:
#> [1] stats     graphics  grDevices utils     datasets  methods   base
#>
#> other attached packages:
#> [1] sf_0.9-6     raster_3.1-5 sp_1.4-4     foot_0.6
#>
#> loaded via a namespace (and not attached):
#>  [1] Rcpp_1.0.5         highr_0.8          pillar_1.4.3       compiler_3.6.3
#>  [5] formatR_1.7        class_7.3-16       iterators_1.0.13   tools_3.6.3
#>  [9] digest_0.6.27      lattice_0.20-41    evaluate_0.14      lifecycle_0.2.0
#> [13] tibble_3.0.1       pkgconfig_2.0.3    rlang_0.4.8        foreach_1.5.1
#> [17] DBI_1.1.0          filelock_1.0.2     rgdal_1.4-8        yaml_2.2.1
#> [21] parallel_3.6.3     xfun_0.18          e1071_1.7-4        stringr_1.4.0
#> [25] dplyr_1.0.2        knitr_1.30         generics_0.0.2     vctrs_0.3.4
#> [29] tidyselect_1.1.0   classInt_0.4-3     grid_3.6.3         glue_1.4.2
#> [33] data.table_1.13.2  R6_2.5.0           rmarkdown_2.1      purrr_0.3.4
#> [37] magrittr_1.5       stars_0.4-3        codetools_0.2-16   htmltools_0.4.0
#> [41] ellipsis_0.3.1     units_0.6-7        abind_1.4-5        KernSmooth_2.23-16
#> [45] stringi_1.4.6      doParallel_1.0.16  lwgeom_0.2-5       crayon_1.3.4
```

Using custom summary functions in `foot`

December 10, 2020

The `foot` package was developed by WorldPop at the University of Southampton (www.worldpop.org) to support geometric calculations and summaries of measures from building footprint polygons. This vignette demonstrates how users can extend the basic functionality of `calculate_footstats` and `calculate_bigfoot` by making and supplying their own functions to summarise footprint characteristics. For an introduction to the package, see `vignette("footsteps")`.

```
library(foot)
```

Calculations with `foot`

As noted in the introductory vignettes, `foot` primarily uses `calculate_footstats` to calculate and summarise metrics. Internally this function uses `data.table` in order to handle large sets of building footprints and efficiently summarise them. The attributes to be summarised (`what`) are supplied to function names (`how`). These internal structures also allow for user-defined functions to be specified.

Data preparation

To demonstrate using custom functions, we will first add some additional attribute "data" to the footprints which we will use.

```
data("kampala", package = "foot")

buildings <- kampala$buildings
adminzones <- kampala$adminZones

# Adding random data categorical variable
buildings$category <- sample(LETTERS[1:5], size = nrow(buildings), replace = T)
# continuous variable
buildings$mult <- sample(rnorm(nrow(buildings), mean = 10, sd = 2))
```

We can use any attributes of the footprints within `calculate_footstats` and `calculate_bigfoot`, not only the built-in morphology measures listed by `list_fs(what='all')`.

```
# summarising a new data value
calculate_footstats(buildings,
                    adminzones,
                    what="mult", # new attribute to summarise
                    how="mean",
                    verbose=F)
#>     zoneID mult_mean
#>  1:      1  9.681632
#>  2:      2 10.029829
#>  3:      3 10.215038
#>  4:      4 10.208911
#>  5:      5 10.312505
#>  6:      6  9.974659
#>  7:      7  9.211024
```

```
#>  8:      8  9.988926
#>  9:      9  9.936068
#> 10:     10 10.334259
#> 11:     11  9.955476
#> 12:     12  9.561723
#> 13:     13  9.331411
#> 14:     14 10.238511
#> 15:     15  9.959502
#> 16:     16 10.180857
#> 17:     17 10.039047
#> 18:     18 10.082245
#> 19:     19 10.008071
#> 20:     20  9.989263
#> 21:     21  9.340944
#> 22:     22  9.780840
#> 23:     23 10.237982
#> 24:     24  9.860261
#> 25:     25  9.667409
#> 26:     26  9.937505
#> 27:     27  9.921654
#> 28:     29  9.925844
#> 29:     30 10.069237
#> 30:     31  8.950171
#> 31:     32  9.824925
#> 32:     33 10.359008
#> 33:     34  9.880299
#>     zoneID mult_mean
```

Additional built-in functions

The internal `foot` functions are documented in `?fs_functions`; however, these functions are intended to be used within the wrapper functions of `foot` and are rarely intended to be used as standalone functions. One built-in summary function, not applied by default, is `majority`. It is designed to summarise categorical data. This function is available for users in the same manner of specifying the `how` argument.

```
# get the majority category in each zone
calculate_footstats(buildings,
                    adminzones,
                    what="category",
                    how="majority",
                    verbose=F)
#>     zoneID category_majority
#>  1:      1                 E
#>  2:      2                 A
#>  3:      3                 E
#>  4:      4                 B
#>  5:      5                 C
#>  6:      6                 B
#>  7:      7                 B
#>  8:      8                 A
#>  9:      9                 E
#> 10:     10                 B
#> 11:     11                 A
#> 12:     12                 A
```

```
#> 13:     13                 B
#> 14:     14                 C
#> 15:     15                 B
#> 16:     16                 B
#> 17:     17                 A
#> 18:     18                 A
#> 19:     19                 A
#> 20:     20                 A
#> 21:     21                 C
#> 22:     22                 C
#> 23:     23                 A
#> 24:     24                 E
#> 25:     25                 B
#> 26:     26                 E
#> 27:     27                 E
#> 28:     29                 D
#> 29:     30                 A
#> 30:     31                 A
#> 31:     32                 B
#> 32:     33                 D
#> 33:     34                 B
#>     zoneID category_majority
```

The `majority` function is similar to the idea of supplying a user-defined function which is demonstrated in the next section.

User-defined summary functions

Creating functions for use with `foot` follows the same procedures and syntax for functions in `R` in general. They must be declared with `<- function()` and they must be available within the environment where `foot` functions are being used. When the functions are used internally by `calculate_footstats`, they are applied to footprints by zone index. Therefore they should return a single, summary value since the function for that group of footprints in the zone.

The name of the function is what is passed to `foot` as an argument to `how`. The argument(s) to the custom function can be named anything, but they will typically be values present within the footprint attributes to be summarised.

The example below shows a simple function that calculates the sum of the square root of the values. We will apply it to 'area', and `foot` will automatically pre-calculate this characteristic since it is not present in the column names of the footprints.

```
# simple function example 1
f1 <- function(v){
  units(v) <- NULL # ignore units in our function
  return(sum(sqrt(v)))
}

# applying custom summary function to area
calculate_footstats(buildings,
                    adminzones,
                    what="area", how="f1",
                    verbose=F)
#> Linking to GEOS 3.8.0, GDAL 3.0.4, PROJ 6.3.1
#> WARNING: different compile-time and runtime versions for GEOS found:
```

```
#> Linked against: 3.8.0-CAPI-1.13.1  compiled against: 3.8.1-CAPI-1.13.3
#> It is probably a good idea to reinstall sf, and maybe rgeos and rgdal too
#>     zoneID    area_f1
#>  1:      1  364.18909
#>  2:      2 1823.23957
#>  3:      3  871.63637
#>  4:      4 1966.54994
#>  5:      5  867.92154
#>  6:      6  822.51869
#>  7:      7  485.66087
#>  8:      8 1194.46899
#>  9:      9 3048.19689
#> 10:     10  385.24272
#> 11:     11 1404.13211
#> 12:     12  294.77870
#> 13:     13   73.79837
#> 14:     14  730.25610
#> 15:     15 2014.66636
#> 16:     16 1154.35602
#> 17:     17 1133.49662
#> 18:     18 3472.56857
#> 19:     19 5905.48635
#> 20:     20 5262.66216
#> 21:     21  998.62900
#> 22:     22  722.37587
#> 23:     23 3441.00032
#> 24:     24 3882.17755
#> 25:     25  435.66830
#> 26:     26 3426.43477
#> 27:     27 2283.48433
#> 28:     29 1910.57791
#> 29:     30  989.77450
#> 30:     31   11.92210
#> 31:     32 1913.14204
#> 32:     33  309.58519
#> 33:     34 9839.29584
#>     zoneID    area_f1
```

Although this function was just used to process area, the function can be used for any continuous value. It can also be used on multiple characteristics or combined with other lists of functions, just like any other built-in function in `foot`.

```
calculate_footstats(buildings,
                    adminzones,
                    what=list("area","perimeter"), how="f1",
                    verbose=F)
#>     zoneID    area_f1 perimeter_f1
#>  1:      1  364.18909   183.488651
#>  2:      2 1823.23957  1048.879091
#>  3:      3  871.63637   426.068457
#>  4:      4 1966.54994   968.615609
#>  5:      5  867.92154   412.069737
#>  6:      6  822.51869   337.518569
#>  7:      7  485.66087   171.066602
```

```
#>  8:      8 1194.46899   515.464525
#>  9:      9 3048.19689  1911.775834
#> 10:     10  385.24272   226.579855
#> 11:     11 1404.13211   857.273283
#> 12:     12  294.77870   214.216882
#> 13:     13   73.79837    35.239420
#> 14:     14  730.25610   500.492421
#> 15:     15 2014.66636  1407.986177
#> 16:     16 1154.35602   835.883724
#> 17:     17 1133.49662   716.240827
#> 18:     18 3472.56857  2477.699320
#> 19:     19 5905.48635  3199.202606
#> 20:     20 5262.66216  2994.324208
#> 21:     21  998.62900   552.056838
#> 22:     22  722.37587   400.124081
#> 23:     23 3441.00032  1548.754569
#> 24:     24 3882.17755  2167.419552
#> 25:     25  435.66830   207.129346
#> 26:     26 3426.43477  1360.146927
#> 27:     27 2283.48433  1300.818322
#> 28:     29 1910.57791  1117.788513
#> 29:     30  989.77450   569.928890
#> 30:     31   11.92210     6.938055
#> 31:     32 1913.14204  1042.133260
#> 32:     33  309.58519   155.175160
#> 33:     34 9839.29584  6005.424628
#>     zoneID    area_f1 perimeter_f1
```

Functions with multiple arguments

In some instances, a custom function may need to make use of two or more characteristics from within the building footprint datasets. The built-in functions in `foot` are primarily designed to work with a single value (e.g. area or perimeter).

While it may sometimes be quicker to pre-calculate the combination, it could be advantageous to use a function, particularly in `calculate_bigfoot` where smaller subsets of a large building footprint dataset are processed. To make sure multiple attributes are supplied to the summary function, the arguments in `what` should be specified using a special type (`fs_varlist`). The `fs_varlist` creates a nested list within the internal processing to keep the argument together. Keep in mind that the arguments are passed to the summary function by **position**, not be name, so the order within `fs_varlist` must match the order of parameters that the function is expecting.

Creating a Perimeter/Area ratio

An example of a custom function using two characteristics is the average perimeter-area ratio. We can compare this to the built-in function which uses a Polsby-Popper metric (`fs_compact`).

```
# average perimeter-area ratio
pa <- function(p, a){
  return(mean(p / a))
}

# used to summarise within zones
# note that fs_varlist is still within a list
calculate_footstats(buildings,
```

```r
                    adminzones,
                    what=list(list("compact"), fs_varlist("perimeter","area")),
                    how=list(list("mean"), list("pa")),
                    verbose=T
                   )
#> Selecting metrics
#> Setting control values.
#> Pre-calculating areas
#> Pre-calculating perimeters
#> Pre-calculating compactness
#> Creating zonal index
#>
#> Calculating  2  metrics ...
#>    compact mean
#>    perimeter area pa
#> Finished calculating metrics.
#>     zoneID compact_mean perimeter_area_pa
#>  1:      1    0.5991569   0.3678351 [1/m]
#>  2:      2    0.6575220   0.4310395 [1/m]
#>  3:      3    0.6126862   0.3865439 [1/m]
#>  4:      4    0.6272569   0.3997248 [1/m]
#>  5:      5    0.6053632   0.4032383 [1/m]
#>  6:      6    0.6255586   0.3133090 [1/m]
#>  7:      7    0.5447738   0.2699737 [1/m]
#>  8:      8    0.5516914   0.3056295 [1/m]
#>  9:      9    0.6113972   0.5041525 [1/m]
#> 10:     10    0.6219757   0.5085339 [1/m]
#> 11:     11    0.5777758   0.4693639 [1/m]
#> 12:     12    0.6591031   0.6469146 [1/m]
#> 13:     13    0.5629929   0.5594556 [1/m]
#> 14:     14    0.7122782   0.4881872 [1/m]
#> 15:     15    0.7247100   0.5306499 [1/m]
#> 16:     16    0.6982459   0.5678706 [1/m]
#> 17:     17    0.6518730   0.5087060 [1/m]
#> 18:     18    0.6869924   0.6245742 [1/m]
#> 19:     19    0.6686708   0.3519480 [1/m]
#> 20:     20    0.6810544   0.3832515 [1/m]
#> 21:     21    0.6926037   0.3695641 [1/m]
#> 22:     22    0.7113827   0.3252631 [1/m]
#> 23:     23    0.6151105   0.4001308 [1/m]
#> 24:     24    0.6682211   0.3709533 [1/m]
#> 25:     25    0.6304162   0.3606033 [1/m]
#> 26:     26    0.6118970   0.3036161 [1/m]
#> 27:     27    0.6831782   0.3904080 [1/m]
#> 28:     29    0.6616867   0.4517787 [1/m]
#> 29:     30    0.6448110   0.3991052 [1/m]
#> 30:     31    0.7708405   0.3386647 [1/m]
#> 31:     32    0.6358119   0.3916160 [1/m]
#> 32:     33    0.5847305   0.3451821 [1/m]
#> 33:     34    0.6413189   0.4558177 [1/m]
#>     zoneID compact_mean perimeter_area_pa
```

Accessing R objects other than the footprints

A more complicated scenario exists when a user-defined function needs to access data which is not an attribute of the footprints dataset. In order to access the non-footprint data, a partial function must be created first and then supplied to calculation function.

In the example below, a simple constant value is supplied to a summary function; however, the idea extends to any object in the R environment. This process is how the nearest neighbour index is calculated in `foot` by drawing on the spatial zones object as well as the footprints.

```
# external "data"
d1 <- 0.001

# This will NOT work because argument 'd' is not found
# f2 <- function(x, d){
#   return(sum(d * x))
# }
#
# calculate_footstats(buildings, adminzones, what="area", how="f2", verbose=T)

# Instead...
# example of creating a partial function
gen_f3 <- function(d){
  force(d) # must include
  function(x){
    return(sum(d * x))
  }
}

# generate the function and initialise it with `d1` from above.
f3 <- gen_f3(d1)

# this now uses the generated function, and `d` is found
calculate_footstats(buildings,
                    adminzones,
                    what="area",
                    how="f3",
                    verbose=F
                   )
#>     zoneID          area_f3
#>  1:      1   8.4545661 [m^2]
#>  2:      2  29.7585314 [m^2]
#>  3:      3  25.7286600 [m^2]
#>  4:      4  63.8357113 [m^2]
#>  5:      5  26.7296259 [m^2]
#>  6:      6  35.7683531 [m^2]
#>  7:      7  28.3878563 [m^2]
#>  8:      8  44.5041537 [m^2]
#>  9:      9  48.1457825 [m^2]
#> 10:     10   7.5599164 [m^2]
#> 11:     11  24.2123780 [m^2]
#> 12:     12   3.1098042 [m^2]
#> 13:     13   2.6464756 [m^2]
#> 14:     14   6.7120641 [m^2]
#> 15:     15  19.2269062 [m^2]
```

```
#> 16:     16   9.9002318 [m^2]
#> 17:     17  14.7525754 [m^2]
#> 18:     18  36.8954355 [m^2]
#> 19:     19  96.4900399 [m^2]
#> 20:     20  80.3556287 [m^2]
#> 21:     21  18.0633304 [m^2]
#> 22:     22  10.1969657 [m^2]
#> 23:     23 132.6730618 [m^2]
#> 24:     24  61.8347854 [m^2]
#> 25:     25  11.6430956 [m^2]
#> 26:     26 150.2087190 [m^2]
#> 27:     27  33.8168355 [m^2]
#> 28:     29  37.0078380 [m^2]
#> 29:     30  15.2761021 [m^2]
#> 30:     31   0.1421365 [m^2]
#> 31:     32  35.7328487 [m^2]
#> 32:     33   7.1172864 [m^2]
#> 33:     34 146.9351905 [m^2]
#>     zoneID           area_f3
```

In this vignette, the `foot` package has been extended to incorporate user-defined functions. These functions can use one or more values from within the footprints, or even access other objects in the environment. While the examples used `calculate_footstats`, the same approaches can be used to create new gridded summary metrics with `calculate_bigfoot`.

```
sessionInfo()
#> R version 3.6.3 (2020-02-29)
#> Platform: x86_64-pc-linux-gnu (64-bit)
#> Running under: Ubuntu 20.04.1 LTS
#>
#> Matrix products: default
#> BLAS:   /usr/lib/x86_64-linux-gnu/blas/libblas.so.3.9.0
#> LAPACK: /usr/lib/x86_64-linux-gnu/lapack/liblapack.so.3.9.0
#>
#> locale:
#>  [1] LC_CTYPE=en_GB.UTF-8       LC_NUMERIC=C
#>  [3] LC_TIME=en_GB.UTF-8        LC_COLLATE=en_GB.UTF-8
#>  [5] LC_MONETARY=en_GB.UTF-8    LC_MESSAGES=en_GB.UTF-8
#>  [7] LC_PAPER=en_GB.UTF-8       LC_NAME=C
#>  [9] LC_ADDRESS=C               LC_TELEPHONE=C
#> [11] LC_MEASUREMENT=en_GB.UTF-8 LC_IDENTIFICATION=C
#>
#> attached base packages:
#> [1] stats     graphics  grDevices utils     datasets  methods   base
#>
#> other attached packages:
#> [1] sf_0.9-6 foot_0.6
#>
#> loaded via a namespace (and not attached):
#>  [1] Rcpp_1.0.5         pillar_1.4.3       compiler_3.6.3     formatR_1.7
#>  [5] class_7.3-16       iterators_1.0.13   tools_3.6.3        digest_0.6.27
#>  [9] evaluate_0.14      lifecycle_0.2.0    tibble_3.0.1       pkgconfig_2.0.3
#> [13] rlang_0.4.8        foreach_1.5.1      DBI_1.1.0          filelock_1.0.2
```

```
#> [17] yaml_2.2.1         parallel_3.6.3     xfun_0.18          e1071_1.7-4
#> [21] stringr_1.4.0      dplyr_1.0.2        knitr_1.30         generics_0.0.2
#> [25] vctrs_0.3.4        tidyselect_1.1.0   classInt_0.4-3     grid_3.6.3
#> [29] glue_1.4.2         data.table_1.13.2  R6_2.5.0           rmarkdown_2.1
#> [33] purrr_0.3.4        magrittr_1.5       stars_0.4-3        codetools_0.2-16
#> [37] htmltools_0.4.0    ellipsis_0.3.1     units_0.6-7        abind_1.4-5
#> [41] KernSmooth_2.23-16 stringi_1.4.6      doParallel_1.0.16  lwgeom_0.2-5
#> [45] crayon_1.3.4
```
