# Supplementary material for: Tools for mapping multi-scale settlement patterns of building footprints: An introduction to the R package foot
Source: PLoS One. 2021 Feb 25;16(2):e0247535. doi: 10.1371/journal.pone.0247535 (PMC7906393; doi:10.1371/journal.pone.0247535)

The settlement type classification was derived from gridded building morphometric layers using a Gaussian Mixture Model (GMM) for unsupervised clustering. The classificatioin is applied as a 100 m x 100 m gridded dataset.

Data shown are the authors' calculations using building footprint polygons and boundary layers from Ordnance Survey OS OpenMap - Local (Contains OS data © Crown copyright and database right, 2018, 2020) released under the Open Government Licence (OGL v3.0).

Coordinate reference information: EPSG: 27700

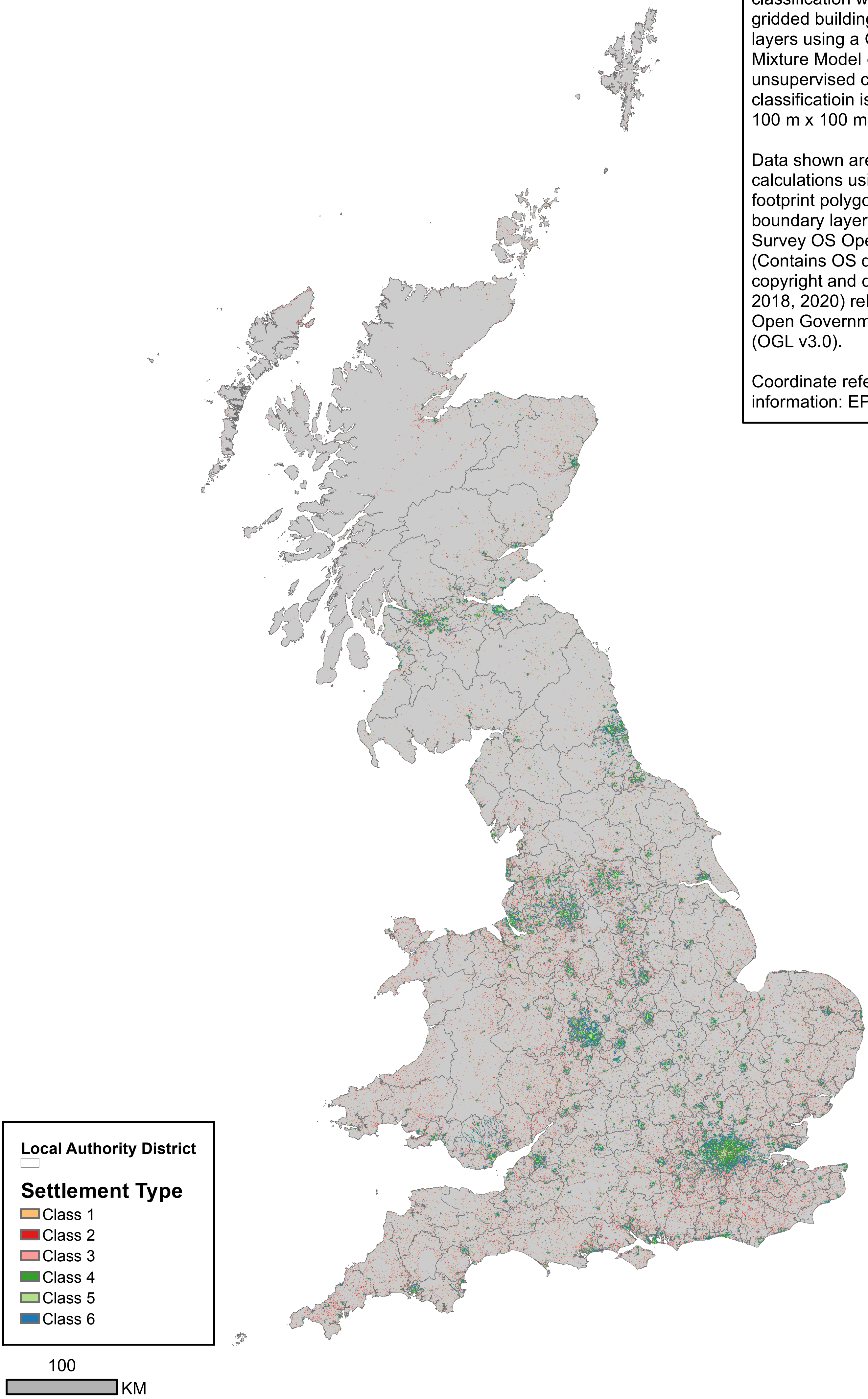

Supplement: S1 File — (PDF) [file pone.0247535.s004.pdf]
