# Supplementary material for: Tools for mapping multi-scale settlement patterns of building footprints: An introduction to the R package foot
Source: PLoS One. 2021 Feb 25;16(2):e0247535. doi: 10.1371/journal.pone.0247535 (PMC7906393; doi:10.1371/journal.pone.0247535)
Supplement: Attachment — Submitted filename: Response to Reviewers.docx [file pone.0247535.s005.docx]

Response to Reviewers

Dear Dr. Yu and Reviewers,

Thank you for your time and attention to reviewing our manuscript. We appreciate your comments and questions and that you have found merit in our work. Below we are addressing each concern in a point-by-point response. From here, our responses to comments will be marked in **red** font. Thank you for your consideration.

Yours sincerely,

Warren Jochem

Additional Editor Comments:

1. Please ensure that your manuscript meets PLOS ONE's style requirements, including those for file naming. The PLOS ONE style templates can be found at

[https://journals.plos.org/plosone/s/file?id=wjVg/PLOSOne_formatting_sample_main_body.pdf](https://eur03.safelinks.protection.outlook.com/?url=https%3A%2F%2Fjournals.plos.org%2Fplosone%2Fs%2Ffile%3Fid%3DwjVg%2FPLOSOne_formatting_sample_main_body.pdf&data=04%7C01%7CW.C.Jochem%40soton.ac.uk%7Cbb30f76f8e4444038f3408d8bbbb68d3%7C4a5378f929f44d3ebe89669d03ada9d8%7C0%7C0%7C637465760870355822%7CUnknown%7CTWFpbGZsb3d8eyJWIjoiMC4wLjAwMDAiLCJQIjoiV2luMzIiLCJBTiI6Ik1haWwiLCJXVCI6Mn0%3D%7C1000&sdata=Fw%2BomMRkDnKt94i1XlvkbpaTnXMeNGfiGU9i7hnfgeE%3D&reserved=0) and [https://journals.plos.org/plosone/s/file?id=ba62/PLOSOne_formatting_sample_title_authors_affiliations.pdf](https://eur03.safelinks.protection.outlook.com/?url=https%3A%2F%2Fjournals.plos.org%2Fplosone%2Fs%2Ffile%3Fid%3Dba62%2FPLOSOne_formatting_sample_title_authors_affiliations.pdf&data=04%7C01%7CW.C.Jochem%40soton.ac.uk%7Cbb30f76f8e4444038f3408d8bbbb68d3%7C4a5378f929f44d3ebe89669d03ada9d8%7C0%7C0%7C637465760870365779%7CUnknown%7CTWFpbGZsb3d8eyJWIjoiMC4wLjAwMDAiLCJQIjoiV2luMzIiLCJBTiI6Ik1haWwiLCJXVCI6Mn0%3D%7C1000&sdata=GCeh7GH5Ji9SvRMK6Ey%2Bw0M5y5epm1yNMa00NPC6%2B9Q%3D&reserved=0)

We have reviewed the style requirements and reformatted our manuscript and our file names, especially the additional figures and figure captions.

As requested in your email, we have submitted our figures for verification through the PACE system prior to uploading them to this revision.

2. We note that you have stated that you will provide repository information for your data at acceptance. Should your manuscript be accepted for publication, we will hold it until you provide the relevant accession numbers or DOIs necessary to access your data. If you wish to make changes to your Data Availability statement, please describe these changes in your cover letter and we will update your Data Availability statement to reflect the information you provide.

We understand this requirement and will provide the necessary DOI numbers to the Editor on acceptance of our manuscript. Our files have already been deposited in a repository through our institution, which will grant the numbers once the manuscript has been accepted. Temporary/placeholder numbers have currently been assigned and our institution is waiting for the manuscript to be accepted.

3. We note that Figures 1-5, Supplementary File 2 (F1, F2), Supplementary File 4 in your submission contain [map/satellite] images which may be copyrighted. All PLOS content is published under the Creative Commons Attribution License (CC BY 4.0), which means that the manuscript, images, and Supporting Information files will be freely available online, and any third party is permitted to access, download, copy, distribute, and use these materials in any way, even commercially, with proper attribution. For these reasons, we cannot publish previously copyrighted maps or satellite images created using proprietary data, such as Google software (Google Maps, Street View, and Earth). For more information, see our copyright guidelines: [http://journals.plos.org/plosone/s/licenses-and-copyright](https://eur03.safelinks.protection.outlook.com/?url=http%3A%2F%2Fjournals.plos.org%2Fplosone%2Fs%2Flicenses-and-copyright&data=04%7C01%7CW.C.Jochem%40soton.ac.uk%7Cbb30f76f8e4444038f3408d8bbbb68d3%7C4a5378f929f44d3ebe89669d03ada9d8%7C0%7C0%7C637465760870365779%7CUnknown%7CTWFpbGZsb3d8eyJWIjoiMC4wLjAwMDAiLCJQIjoiV2luMzIiLCJBTiI6Ik1haWwiLCJXVCI6Mn0%3D%7C1000&sdata=2BRgP6J43ZUyCFURPkFuG0Q%2FFIAfHe01bGyl2TiO6TU%3D&reserved=0).

We require you to either (1) present written permission from the copyright holder to publish these figures specifically under the CC BY 4.0 license, or (2) remove the figures from your submission:

3.1.    You may seek permission from the original copyright holder of Figures 1-5, Supplementary File 2 (F1, F2), Supplementary File 4 to publish the content specifically under the CC BY 4.0 license. We recommend that you contact the original copyright holder with the Content Permission Form ([http://journals.plos.org/plosone/s/file?id=7c09/content-permission-form.pdf](https://eur03.safelinks.protection.outlook.com/?url=http%3A%2F%2Fjournals.plos.org%2Fplosone%2Fs%2Ffile%3Fid%3D7c09%2Fcontent-permission-form.pdf&data=04%7C01%7CW.C.Jochem%40soton.ac.uk%7Cbb30f76f8e4444038f3408d8bbbb68d3%7C4a5378f929f44d3ebe89669d03ada9d8%7C0%7C0%7C637465760870375738%7CUnknown%7CTWFpbGZsb3d8eyJWIjoiMC4wLjAwMDAiLCJQIjoiV2luMzIiLCJBTiI6Ik1haWwiLCJXVCI6Mn0%3D%7C1000&sdata=1QHJO7VNBUJnDSx%2FyHvxsr3BYh8G9of0zIlV5FPlbAk%3D&reserved=0)) and the following text:

“I request permission for the open-access journal PLOS ONE to publish XXX under the Creative Commons Attribution License (CCAL) CC BY 4.0 ([http://creativecommons.org/licenses/by/4.0/](https://eur03.safelinks.protection.outlook.com/?url=http%3A%2F%2Fcreativecommons.org%2Flicenses%2Fby%2F4.0%2F&data=04%7C01%7CW.C.Jochem%40soton.ac.uk%7Cbb30f76f8e4444038f3408d8bbbb68d3%7C4a5378f929f44d3ebe89669d03ada9d8%7C0%7C0%7C637465760870375738%7CUnknown%7CTWFpbGZsb3d8eyJWIjoiMC4wLjAwMDAiLCJQIjoiV2luMzIiLCJBTiI6Ik1haWwiLCJXVCI6Mn0%3D%7C1000&sdata=mnrXS4dKndtG%2Fi4Mt7I3%2FfGHB42HYefJb9Di0wly%2Fo0%3D&reserved=0)). Please be aware that this license allows unrestricted use and distribution, even commercially, by third parties. Please reply and provide explicit written permission to publish XXX under a CC BY license and complete the attached form.”

Please upload the completed Content Permission Form or other proof of granted permissions as an "Other" file with your submission.

In the figure caption of the copyrighted figure, please include the following text: “Reprinted from [ref] under a CC BY license, with permission from [name of publisher], original copyright [original copyright year].”

3.2.    If you are unable to obtain permission from the original copyright holder to publish these figures under the CC BY 4.0 license or if the copyright holder’s requirements are incompatible with the CC BY 4.0 license, please either i) remove the figure or ii) supply a replacement figure that complies with the CC BY 4.0 license. Please check copyright information on all replacement figures and update the figure caption with source information. If applicable, please specify in the figure caption text when a figure is similar but not identical to the original image and is therefore for illustrative purposes only.

The following resources for replacing copyrighted map figures may be helpful:

USGS National Map Viewer (public domain): [http://viewer.nationalmap.gov/viewer/](https://eur03.safelinks.protection.outlook.com/?url=http%3A%2F%2Fviewer.nationalmap.gov%2Fviewer%2F&data=04%7C01%7CW.C.Jochem%40soton.ac.uk%7Cbb30f76f8e4444038f3408d8bbbb68d3%7C4a5378f929f44d3ebe89669d03ada9d8%7C0%7C0%7C637465760870385691%7CUnknown%7CTWFpbGZsb3d8eyJWIjoiMC4wLjAwMDAiLCJQIjoiV2luMzIiLCJBTiI6Ik1haWwiLCJXVCI6Mn0%3D%7C1000&sdata=Hk4NG1H3gqquheks28q7qM%2BuWKkI46w4vWYnrr5cS6g%3D&reserved=0)

The Gateway to Astronaut Photography of Earth (public domain): [http://eol.jsc.nasa.gov/sseop/clickmap/](https://eur03.safelinks.protection.outlook.com/?url=http%3A%2F%2Feol.jsc.nasa.gov%2Fsseop%2Fclickmap%2F&data=04%7C01%7CW.C.Jochem%40soton.ac.uk%7Cbb30f76f8e4444038f3408d8bbbb68d3%7C4a5378f929f44d3ebe89669d03ada9d8%7C0%7C0%7C637465760870385691%7CUnknown%7CTWFpbGZsb3d8eyJWIjoiMC4wLjAwMDAiLCJQIjoiV2luMzIiLCJBTiI6Ik1haWwiLCJXVCI6Mn0%3D%7C1000&sdata=5deLxL0dvsl%2BCVOzjp1Oao2Qfybb8iToh4ChcoWP%2Ffk%3D&reserved=0)

Maps at the CIA (public domain): [https://www.cia.gov/library/publications/the-world-factbook/index.html](https://eur03.safelinks.protection.outlook.com/?url=https%3A%2F%2Fwww.cia.gov%2Flibrary%2Fpublications%2Fthe-world-factbook%2Findex.html&data=04%7C01%7CW.C.Jochem%40soton.ac.uk%7Cbb30f76f8e4444038f3408d8bbbb68d3%7C4a5378f929f44d3ebe89669d03ada9d8%7C0%7C0%7C637465760870395649%7CUnknown%7CTWFpbGZsb3d8eyJWIjoiMC4wLjAwMDAiLCJQIjoiV2luMzIiLCJBTiI6Ik1haWwiLCJXVCI6Mn0%3D%7C1000&sdata=m4kzEdGXQJP6HR8V6I%2B%2Bode0%2FbJ3qYmN3mmKYD3ufQU%3D&reserved=0) and [https://www.cia.gov/library/publications/cia-maps-publications/index.html](https://eur03.safelinks.protection.outlook.com/?url=https%3A%2F%2Fwww.cia.gov%2Flibrary%2Fpublications%2Fcia-maps-publications%2Findex.html&data=04%7C01%7CW.C.Jochem%40soton.ac.uk%7Cbb30f76f8e4444038f3408d8bbbb68d3%7C4a5378f929f44d3ebe89669d03ada9d8%7C0%7C0%7C637465760870395649%7CUnknown%7CTWFpbGZsb3d8eyJWIjoiMC4wLjAwMDAiLCJQIjoiV2luMzIiLCJBTiI6Ik1haWwiLCJXVCI6Mn0%3D%7C1000&sdata=lHQxigK4ndXftWJ%2FpUSghCR0b1OMT4gvn6FkwfwiWYw%3D&reserved=0)

NASA Earth Observatory (public domain): [http://earthobservatory.nasa.gov/](https://eur03.safelinks.protection.outlook.com/?url=http%3A%2F%2Fearthobservatory.nasa.gov%2F&data=04%7C01%7CW.C.Jochem%40soton.ac.uk%7Cbb30f76f8e4444038f3408d8bbbb68d3%7C4a5378f929f44d3ebe89669d03ada9d8%7C0%7C0%7C637465760870405607%7CUnknown%7CTWFpbGZsb3d8eyJWIjoiMC4wLjAwMDAiLCJQIjoiV2luMzIiLCJBTiI6Ik1haWwiLCJXVCI6Mn0%3D%7C1000&sdata=IgrxJf8%2Fk40tea%2Fv086%2BFo8x11ZGNcuYHKybrKOFceE%3D&reserved=0)

Landsat: [http://landsat.visibleearth.nasa.gov/](https://eur03.safelinks.protection.outlook.com/?url=http%3A%2F%2Flandsat.visibleearth.nasa.gov%2F&data=04%7C01%7CW.C.Jochem%40soton.ac.uk%7Cbb30f76f8e4444038f3408d8bbbb68d3%7C4a5378f929f44d3ebe89669d03ada9d8%7C0%7C0%7C637465760870405607%7CUnknown%7CTWFpbGZsb3d8eyJWIjoiMC4wLjAwMDAiLCJQIjoiV2luMzIiLCJBTiI6Ik1haWwiLCJXVCI6Mn0%3D%7C1000&sdata=X1%2BNF4FkBh5498iHHCCtm51aAkk1x7Qu8PnAxGylKhI%3D&reserved=0)

USGS EROS (Earth Resources Observatory and Science (EROS) Center) (public domain): [http://eros.usgs.gov/#](https://eur03.safelinks.protection.outlook.com/?url=http%3A%2F%2Feros.usgs.gov%2F%23&data=04%7C01%7CW.C.Jochem%40soton.ac.uk%7Cbb30f76f8e4444038f3408d8bbbb68d3%7C4a5378f929f44d3ebe89669d03ada9d8%7C0%7C0%7C637465760870415562%7CUnknown%7CTWFpbGZsb3d8eyJWIjoiMC4wLjAwMDAiLCJQIjoiV2luMzIiLCJBTiI6Ik1haWwiLCJXVCI6Mn0%3D%7C1000&sdata=PBMla300%2BQzSAk8VtAMyd5x%2Fyvzd8pW6LdCd7KuNy98%3D&reserved=0)

Natural Earth (public domain): [http://www.naturalearthdata.com/](https://eur03.safelinks.protection.outlook.com/?url=http%3A%2F%2Fwww.naturalearthdata.com%2F&data=04%7C01%7CW.C.Jochem%40soton.ac.uk%7Cbb30f76f8e4444038f3408d8bbbb68d3%7C4a5378f929f44d3ebe89669d03ada9d8%7C0%7C0%7C637465760870415562%7CUnknown%7CTWFpbGZsb3d8eyJWIjoiMC4wLjAwMDAiLCJQIjoiV2luMzIiLCJBTiI6Ik1haWwiLCJXVCI6Mn0%3D%7C1000&sdata=F4tnaVwTw878pVDhg2q6NZ2kjHk1UKhWFVNxhJVLk%2FU%3D&reserved=0)

Thank you for directing us to the policies and resources regarding licensing of images and map data in PLOS One. This manuscript uses only open data, licensed with the Open Government License version 3.0 (<http://www.nationalarchives.gov.uk/doc/open-government-licence/version/3/>) and the Open Database License (<https://opendatacommons.org/licenses/odbl/> ). These licenses are, to our understanding, compatible with CC-BY, allowing copying, re-distribution, adaptation, and re-use (including commercial uses). They are attribution-only licenses which meets the requirement stated in section 3.2 above.

We have amended the manuscript to make the licensing and data sources clear in several places. First, in the main text (lines 137-138, lines 322-323) we have added a note on the source of the data when describing the examples and case study. Second, in each figure caption using these data, we have added text giving the specific data source, copyright and license information.

Reviewers' comments:

Reviewer #1: This is an interesting approach to mapping, and I believe it will achieve positive results in locations where large-scale planning has not been implemented. Countries with limited data and mapping capabilities will benefit from this research.

We thank the reviewer for their time in reviewing our manuscript.

Reviewer #2: Overall the manuscript is well-written and sufficiently descriptive. The R package described appears to be a useful contribution for a variety of research and applications. I only have a few requests before recommending for publication:

We thank the reviewer for their helpful comments and suggestions.

- Please provide a description of morphology parameters that are not obvious. Specifically shape and compactness. Even if these are exhaustively described in cited literature, a brief description should be included here.

We have amended the manuscript to include a more details of the morphology metrics we use. On lines 156 – 162 we have added text describing the shape and compactness measures as well as the nearest neighbour index in lines 180 – 181. These additions include the questions, describing the potential range of values as well as added references to where the measures were developed. As noted below, we have also expanded our explanation of the entropy measure.

- Line 153 - provide an equation for how entropy is calculated in this work

In addition to providing a fuller explanation for the morphology metrics discussed above, we have added the specific equations for entropy and other calculations implemented in our R package (lines 171 – 173). For this work we use Shannon entropy.

- Expand the conclusions to summarize findings from comparison of the clustering effort demonstrated in this paper with the Census and MODUM data described in the paper.

Thank you for this suggestion. We have expanded our discussion section to summarise the findings of our comparisons and to provide more context to the interpretation of our results. Specifically we note that our footprint-derived clusters are consistent with the urban-rural classification gradients and less similar to the MODUM data. This finding is not surprising given that features related to physical form are important in the census classification. However, MODUM draws on additional data sources, and it’s important to consider that such requirements may limit its wider application. A simpler typology deriving solely from footprint patterns (as we demonstrated) could be implemented in more areas. We incorporated these edits into the original discussion section, but the additions are primarily in lines 476 – 514 of the revised manuscript.

- Line 254 - what is meant by proxy datasets?

Thank you for raising this question. We were referring to something like a “file pointer” as the program only requires the location of the input footprint data and does not have to read the entire contents of large files. We agree that wording was unclear and have changed the manuscript. In line 284 of the revised manuscript we have removed the phrase “proxy datasets” as we feel the sentence still conveys the important information that only sub-datasets are extracted and analysed, potentially in parallel.

- Line 239 - change "be" to "been"

We have made this change and reviewed the manuscript again for any other grammar and spelling errors. Thank you.
